# Pharmacological induction of acetyl-CoA carboxylase 1 autophagic degradation attenuates lipid accumulation and cholangiocarcinoma progression

**DOI:** 10.1186/s13046-025-03564-8

**Published:** 2025-11-25

**Authors:** Yani Pan, Nannan Zhang, Xueni Fu, Xinyu Wang, Yichun Ma, Qi Chen, Yue Zhou, Hongwen Liu, Yun Zhu, Lei Xu, Qiang Wang, Dongyin Chen, Zhangding Wang, Lei Wang

**Affiliations:** 1 Department of Gastroenterology, Nanjing Drum Tower Hospital, Nanjing Drum Tower Hospital Clinical College of Nanjing University of Chinese Medicine, Nanjing, 210000 China; 2 Department of Pharmacy, Shidong Hospital, Yangpu District, Shanghai, 200438 China; 3 Department of Gastroenterology, Nanjing Drum Tower Hospital, Drum Tower Clinical Medical College of China Pharmaceutical University, Nanjing, 210000 China; 4 Department of Pathology, Nanjing Drum Tower Hospital, Affiliated Hospital of Medical School, Nanjing University, Nanjing, 210000 China; 5 Department of Gastroenterology, Nanjing Drum Tower Hospital, Affiliated Hospital of Medical School, Nanjing University, Nanjing, Jiangsu Province 210000 People’s Republic of China; 6 Department of Pharmacy, Nanjing Drum Tower Hospital, Affiliated Hospital of Medical School, Nanjing University, Nanjing, 210000 China; 7 Department of Hepatobiliary Surgery, MOE Innovation Center for Basic Research in Tumor Immunotherapy, Anhui Province Key Laboratory of Tumor Immune Microenvironment and Immunotherapy, The First Affiliated Hospital of Anhui Medical University, Hefei, Anhui Province 230000 People’s Republic of China; 8 International Joint Laboratory for Drug Target of Critical Illnesses, School of Pharmacy, Nanjing Medical University, Nanjing, Jiangsu Province 210000 People’s Republic of China

**Keywords:** Cholangiocarcinoma, Lipid metabolism, Withaferin A, Acetyl coenzyme A carboxylase 1

## Abstract

**Background:**

Aberrant glycogen metabolism drives lipid accumulation and adaptive lipid homeostasis reprogramming, a metabolic adaptation critical for sustaining malignant progression and chemoresistance in cholangiocarcinoma (CCA). While our prior study highlighted glycogen degradation as pivotal for CCA tumorigenesis, the molecular mechanisms governing lipogenesis and its therapeutic exploitation remain elusive.

**Methods:**

We performed single-cell RNA sequencing to explore metabolic status in CCA. A high-throughput screening of 994 bioactive compound library was performed to identify pharmacological agents capable of inhibiting CCA and targeting this metabolic vulnerability. The drug efficacy was demonstrated through in vitro and in vivo experiments. Additionally, a biotinylated WA derivative was synthesized and its target was investigated using liquid chromatography-tandem mass spectrometry. Validating the clinical potential of the compound for targeted antitumor therapy in combination with gemcitabine in vivo*.*

**Result:**

Through integrated multi-omics analysis, we identified pronounced lipid droplet accumulation in CCA tissues. Subsequent high-throughput screening of bioactive compounds revealed Withaferin A (WA) as a potent dual suppressor of lipid deposition and cholangiocarcinogenesis. Mechanistically, WA directly binds acetyl-CoA carboxylase 1 (ACC1), inhibiting its catalytic conversion of acetyl-CoA to malonyl-CoA. Notably, resultant malonyl-CoA depletion abolished ACC1 auto-malonylation, thereby enhancing SQSTM1/p62-mediated cargo recognition and triggering selective autophagic degradation, consequently disrupting *de novo* lipogenesis and lipid droplet accumulation. Therapeutically, WA synergized with gemcitabine to enhance antitumor efficacy and prolong survival in preclinical models.

**Conclusion:**

Our study confirms that pharmacological blockade of ACC1 significantly inhibits *de novo* lipogenesis and CCA tumorigenesis, suggesting that WA may serve as a potential small-molecule inhibitor targeting lipid metabolism for CCA treatment.

**Supplementary Information:**

The online version contains supplementary material available at 10.1186/s13046-025-03564-8.

## Introduction

Cholangiocarcinoma (CCA) is a highly aggressive malignancy arising from the biliary epithelium, poses an escalating global health concern with steadily rising incidence rates worldwide [1]. Anatomical complexity, insidious onset, and profound chemoresistance contribute to its characteristically poor prognosis [2, 3]. Therapeutically, current first-line therapy combining gemcitabine and cisplatin, as well as emerging targeted agents (e.g., FGFR inhibitors), show promise in molecularly defined subsets, their overall therapeutic impact remains constrained by intrinsic tumor heterogeneity and acquired mutations [4, 5]. These unmet needs underscore the urgency to delineate fundamental molecular drivers of CCA pathogenesis and develop innovative therapeutic strategies.

Metabolically, glycogen metabolism and lipogenesis constitute evolutionarily conserved mechanisms for cellular energy homeostasis, with their dysregulation emerging as a hallmark of hepatobiliary malignancies [6, 7]. Intriguingly, tumor cores exhibit a paradoxical metabolic feature characterized by concurrent glycogen degradation and lipid droplet accumulation [8]. Our previous study demonstrated that PYGB (Glycogen Phosphorylase B)-driven glycogen degradation sustains the Warburg effect through activation of the pentose phosphate pathway (PPP), generating NADPH to counteract oxidative stress [9]. However, under hypoxic conditions in CCA cells, this protective mechanism becomes maladaptive, as G6PD hyperactivity paradoxically induces oxidative stress. Concurrently, *de novo* lipogenesis intrinsically generates oxidative stress through mitochondrial β-oxidation-derived ROS [10]. Notably, it was indicated that glycogen-derived carbons can be redirected into lipogenic pathways via AMPK inactivation and SREBP1 activation, indicating metabolic crosstalk between these processes [11]. These findings strongly support the notion that aberrant lipid metabolism may constitute an adaptive response to glycogen metabolic dysfunction. However, the precise molecular mechanisms governing lipid droplet accumulation in CCA remain elusive.

Lipid droplets accumulation in malignancies arises from the dysregulated interplay of *de novo* lipogenesis, β-oxidation, and lipid droplet dynamics [12]. Currently, therapeutic strategies disrupting the plasticity of these nodes through enzymatic activity modulation, synthetic lethality and post-translational degradation. For instance, direct catalytic inhibition of fatty acid synthase (FASN) with TVB-2640 suppresses lipogenesis [13], while synthetic lethality approaches like dual ATP-citrate lyase (ACLY)/stearoyl-CoA desaturase 1 (SCD1) inhibition exploit metabolic vulnerabilities [14]. Small-molecule compound like dehydrocostus lactone modulate post-translational states by inhibiting IKKβ-dependent ACLY phosphorylation to trigger its proteasomal degradation [15]. These studies underline the importance of targeting lipid homeostasis, whereas CCA-specific lipid metabolic vulnerabilities remain poorly defined.

In this study, through single-cell RNA sequencing (scRNA-seq) analysis and histological validation, we observed significant lipid droplet accumulation in CCA tissues. Subsequent high-throughput screening of 994 bioactive compounds revealed that Withaferin A (WA) as an effective agent in reducing lipid droplet accumulation and simultaneously inhibiting cholangiocarcinogenesis. Mechanistically, WA directly bound to acetyl-CoA carboxylase 1 (ACC1), inhibiting the catalytic conversion of acetyl-CoA to malonyl-CoA. Notably, the WA-induced depletion of malonyl-CoA abolished the auto-malonylation modification of ACC1, thereby enhancing SQSTM1/p62-mediated recognition and facilitating its lysosomal degradation, ultimately disrupting *de novo* lipid synthesis in CCA cells. Furthermore, both in vitro and in vivo experiments have demonstrated that the combination of WA with gemcitabine synergistically enhances anti-tumor efficacy. Collectively, our study confirmed that ACC1 pharmacological blockade significantly inhibits *de novo* fatty acid synthesis and CCA tumorigenesis, suggesting that WA may as a first-in-class targeted agent disrupting lipid metabolic rewiring for CCA treatment.

## Materials and methods

### Cell lines

The CCA cell line HuCCT1 was obtained from JCRB in Osaka, Japan. The CCA cell lines RBE and HCCC-9810 were purchased from the Institute of Biochemistry and Cell Biology, Shanghai Institutes for Biological Sciences, Chinese Academy of Sciences, Shanghai, China. All cells were cultured in RPMI 1640 medium (Gibco, CA, USA), containing 10% Fetal bovine serum (FBS; Gibco, CA, USA), penicillin (100 U/mL), and streptomycin (100 µg/mL) (Invitrogen, Waltham, MA, USA) at 37 °C in humidity of 5% CO_2_. Cells were identified by short tandem repeat profiling, finally repeated in August 2022. Mycoplasma infection was tested by the MycAway Plus-Color One-Step Mycoplasma Detection Kit (Yeasen, China).

### Human cholangiocarcinoma specimens

A total of five CCA tissue samples were collected from patients at the Nanjing Drum Tower Hospital (Nanjing, Jiangsu, China). All subjects provided informed consent for their participation in this study, which was approved by the Medical Research Ethics Committee of Nanjing Drum Tower Hospital.

### Animal models

Female C57BL/6J mice and BALB/c nude mice (5–6 weeks old) were purchased from GemPharmatech (Jiangsu, China) and housed in a specific pathogen-free (SPF) environment.

For generating CCA mouse models, pT3-EF1α-HA-myr-AKT and pT3-EF1α-YAPS127A plasmids, or KRASG12D and sgP19 plasmids were injected with Sleeping Beauty transposase (SB) via hydrodynamic injection (*n* = 8 per group), as previously reported [9]. Seven days after injection, mice were treated with WA (2 mg/kg or 5 mg/kg, every day) via intraperitoneal injection (MCE, Shanghai, China). After four weeks, the mice were sacrificed and tumor tissues were collected. The treatment regimen for the combination therapy of WA and gemcitabine was administered as described above.

For the xenograft model, a total of 2 × 10^6^ HuCCT1 cells were subcutaneously injected into the axilla of nude mice (*n* = 6 per group). Tumor volumes were monitored (volume = length × width^2^ × 1/2). Ten days after injection, the mice were treated with WA (2 mg/kg or 5 mg/kg, every day). After four weeks, the mice were sacrificed. All tumors were fixed in 4% paraformaldehyde or frozen at −80 °C. The treatment protocol for the combination therapy of WA and rapamycin was carried out as previously described. All animal experiments were approved by the Ethical Committee and Animal Welfare Committee of Nanjing Drum Tower Hospital.

### Oil red O staining and bodipy 493/503 staining

Cells were treated with 200 µM OA (Sigma, MO, USA) for 24 h. For oil Red O staining: oil Red O powder (Sigma, MO, USA) was dissolved in distilled water at a ratio of 3:2, filtered, and prepared as a working solution. Samples were then incubated in 60% isopropanol for 15 min, followed by staining with the oil Red O working solution for 10 min and counterstaining with hematoxylin. For BODIPY 493/503 staining: 100 µL of 10 µM BODIPY 493/503 working solution (MCE, NJ, USA) was added to each well and incubated for 30 min. The nuclei were then stained with DAPI and images were captured using a Leica microscope (Lipid droplet Count = Total number of Lipid droplet ​/Total number of cells).

### CCK-8 assay

Cell viability was assessed using the Cell Counting Kit-8 (CCK-8; Abbkine Scientific Co., Ltd, Wuhan, China) assay according to the manufacturer’s protocol with minor modifications. Briefly, cells were seeded in 96-well plates at 5,000 cells per well. After 24 h cells were treated with test compounds or vehicle control for 24–48 h. Subsequently, 100 µL of serum-free medium containing 10% CCK-8 reagent was added to each well and plates were incubated for 2 h at 37 °C. Absorbance was measured at 450 nm.

### Colony formation assay

After treatment under different conditions, cells were trypsinized and seeded at a density of 1,000 cells per well in a six-well plate, followed by incubation for 2 weeks. Cells were then fixed with 1 mL of methanol for 15 min and stained with crystal violet (Beyotime, Shanghai, China) for 10 min to assess colony formation. Colonies containing more than 50 cells were counted for quantification.

### Transwell assay

Cell migration and invasion were assessed using Transwell chambers (Corning, MA, USA) as previously reported [16]. For the invasion assay, the upper chamber was coated with 100 µL of Matrigel Matrix (Corning, MA, USA) diluted 1:9 in cold serum-free RPMI-1640 and incubated at 37 °C for 1 h to polymerize. Cells were starved in serum-free RPMI-1640 for 24 h, harvested, and resuspended at 5 × 10⁵ cells/mL in serum-free RPMI-1640. Then, 200 µL cell suspension (1 × 10⁵ cells) was added to the upper chamber. The lower chamber contained 600 µL RPMI-1640 with 20% FBS as chemoattractant. After incubated for 24 h, non-migrated/non-invaded cells on the upper membrane surface were removed with a cotton swab. Migrated/invaded cells on the lower surface were fixed with 4% PFA, stained with 0.1% crystal violet (30 min, RT), and washed. Membranes were excised, mounted, and migrated/invaded cells were counted manually in five random fields per membrane at 200x magnification using a light microscope.

### Apoptosis assay

Adherent and supernatant cells were collected after different treatments. Resuspended in 500 µL binding buffer, and gently mixed with 5 µL Annexin V-FITC and 5 µL propidium iodide (KeyGen, Nanjing, China) according to the manufacturer’s protocol. The samples were incubated for 10 min in the dark at room temperature, followed by flow cytometric analysis.

### TUNEL assay

Apoptosis was assessed using the TUNEL Apoptosis Detection Kit (Abbkine Scientific Co., Ltd, Wuhan, China), and cell nuclei were stained with DAPI. Confocal images of the cells were captured using a Leica Thunder Imager microscope system, and TUNEL-positive cells were quantified.

### Western blot

Cell samples were lysed on ice for 15 min in RIPA lysis buffer (Beyotime, Shanghai, China) mixed with protease and phosphatase inhibitor cocktail and phenylmethylsulfonyl fluoride (PMSF) (KeyGen, Nanjing, China). The protein concentration was determined by the BCA assay kit (KeyGen, Nanjing, China). Then, the proteins were subjected to Western blotting according to standard protocols. The primary antibodies used were as follows: ACC1 (21923-1-AP, Proteintech); Cleaved-PARP (9548T, CST); Bcl-2 (12789-1-AP, Proteintech); ATG7 (10088-2-AP, Proteintech); Beclin-1 (11306-1-AP, Proteintech); BNIP3L (12986-1-AP, Proteintech); p62 (18420-1-AP, Proteintech); Tollip (11315-1-AP, Proteintech); NDP52 (12229-1-AP, Proteintech); NRP (10837-1-AP, Proteintech); M17S2 16004-1-AP, Proteintech); β-actin (ab8226, Abcam); Malonylysine (PTM-901, PTM BioLab, Inc.); Acetyllysine (PTM-101, PTM BioLab, Inc.); Crotonyllysine (PTM-501, PTM BioLab, Inc.); Succinyllysine (PTM-401, PTM BioLab, Inc.)

### Immunofluorescence (IF)

The specific procedure for IF is as previously reported [17]. Briefly, cells from different treatments were cultured in confocal dishes, sequentially fixed in 4% PFA, permeabilized with 0.3% Triton X-100 for 10 min, blocked with 5% BSA for 30 min at room temperature, and then incubated with anti-ACC1 (Proteintech) and anti-p62 (Proteintech) primary antibodies at 4 °C overnight. Then rinsed the sample with PBS and incubated with secondary antibodies for 1 h at room temperature. Finally, the cells were stained with DAPI. All images were captured by confocal microscopy.

### Plasmids and siRNA transfection

The specific procedure for plasmids and siRNA transfection is as previously reported [18]. ACC1 mutant plasmids were constructed by Corues Biotechnology (Nanjing, Jiangsu, China), and plasmids were transfered into cells with Lipofectamine 3000 reagent (Invitrogen, USA). 24 h later, the plasmids were removed, and a new culture medium was added. siRNAs targeting ATG7, Beclin-1 and p62 were designed and synthesized by Corues Biotechnology and transfected into cells using Lipofectamine 3000 reagent. The following RNA oligonucleotides were used in this study: siRNA-ctrl forward: 5′- UUCUCCGAACGUGUCACGUTT-3′; Reverse: 5′- ACGUGACACGUUCGGAGAATT-3′; P62 siRNA forward: 5′- GCAUUGAAGUUGAUAUCGA-3′; Reverse: 5′- UCGAUAUCAACUUCAAUGC-3′; Beclin-1 siRNA forward: 5′- GACUUGUUCCUUACGGAAA-3′; Reverse: 5′- UUUCCGUAAGGAACAAGUC-3′; ATG7 siRNA: 5′- GGAGUCACAGCUCUUCCUUAC-3′.

### Real-time quantitative PCR

Total RNA was isolated from the cells using TRIzol reagent (Invitrogen, USA). RNA was reverse transcribed into cDNA using Reverse Transcription Kit (Vazyme, Nanjing, China). Real-time quantitative PCR was performed with ChamQ SYBR Color qPCR Master Mix (Vazyme, Nanjing, China) according to the manufacturer’s instructions. PCR analysis assay was performed using the 2×Taq PCR StarMix (GenStar). The following primers were used: ACC1-F: 5′- TCCGCAGCAATAAGAATGT-3′; ACC1-R: 5′-T CCCCAAGAAAAGCAGTGA-3′; β-actin-F: 5′- TCTCCCAAGTCCACACAGG-3′; β-actin-R: 5′- GGCACGAAGGCTCATCA-3′.

### Synthesis and identification of biotinylated WA

To a solution of WA (50 mg, 0.1mmol) and EDCI (27 mg, 0.14 mmol) in DMF (10 mL). Until the reaction was stirred 30 min at 60 °C temperature, added the Biotin (30 mg, 0.12mmol) and DMAP (2.4 mg, 0.02mmol) to the solution, at the same time, the reaction was stirred until completion (monitored by TLC). The reaction mixture was added water (20 mL), and extracted by DCM (10 mL × 3). The organic layer was separated, washed with brine, and dried over anhydrous Na_2_SO_4_. The solvent was evaporated to dryness, and the residue was purified by flash chromatography (dichloromethane/methanol, 100:1 to 50:1) to give the desired product biotin-WA.

### Immunoprecipitation of biotinylated WA

HuCCT1 cells were cultured to 80% confluence and lysed in IP lysis buffer (Beyotime, Shanghai, China) containing Cocktail and PMSF. After incubation at 4 °C for 1 h, the cells were collected by scraping and centrifuged (12,000 rpm, 20 min, at 4 °C). The supernatant was incubated overnight at 4 °C with biotin-WA or biotin. Then add Streptavidin magnetic beads (Beyotime, Shanghai, China) into the samples and the mixture was rotated for 20 min at room temperature or overnight at 4 °C. The beads were washed with TBST, proteins were eluted by adding 1 x loading buffer and heating. The LC-MS/MS analysis was performed by Bioprofile Biotechnology Co., Ltd. (Shanghai, China).

### Surface plasmon resonance (SPR) assay

SPR assays were performed using a Reichert SR7500DC SPR instrument (Reichert Technologies, Buffalo, NY). Purified ACC1 protein, diluted to 20 µg/mL in 50 mM sodium acetate buffer, was immobilized on a CM5 sensor chip at a flow rate of 30 µL/min. Compounds at specified concentrations were injected into calcium and magnesium-free DPBS buffer containing 5% DMSO at a flow rate of 30 µL/min. Binding for 120 s, and dissociation for 180 s. Results were corrected using the solvent correction method, and all datas were normalized by subtracting the 0 concentration to generate the final binding and dissociation curves.

### Prediction of potential allosteric sites of ACC1 and molecular docking

The ACC1 protein (Uniprot ID: Q13085) was prepared by ADFRsuite-1.0, preserving native charges to generate a pdbqt file. The ligand (CAS: 5119-48−2) was obtained from the CAS database, converted to a 3D structure using OpenBabel, and energy-minimized using the MMFF94 force field by steepest descent optimization. Molecular docking was performed using AutoDock Vina in global search mode.

### Drug affinity responsive target stability (DARTS) assay

Proteins were extracted from CCA cells using IP lysis buffer and quantified by the BCA assay kit (KeyGEN BioTECH, Co., Ltd, Jiangsu, China). Samples were adjusted to 4–6 µg/µL with lysis buffer. After incubation for 1 h at room temperature or overnight at 4 °C, pronase (Merck, USA) was added at a ratio optimized for selective proteolysis (30 min at room temperature) and the reaction was terminated with loading buffer. The processed samples were analyzed by Western blot.

### Cellular thermal shift assay (CETSA)

Cells were treated with WA or DMSO for 1 h at 37 °C, followed by heating (37–62 °C gradient, 3 min) and rapid cooling. Then, cells were lysed in IP lysis buffer, centrifuged (12,000 rpm, 20 min), and supernatants were quantified by the BCA assay kit. The processed samples were analyzed by Western blot.

### Lipidomics analysis

HuCCT1 cells (1 × 10⁶ cells per replicate, triplicates) were treated with WA (2 µM) or DMSO for 24 h, then washed with PBS, centrifuged (1,500 × g, 10 min, 4 °C), and snap-frozen in liquid nitrogen using cryovials pre-chilled with dry ice. Samples were transported on dry ice at temperatures < −70 °C for further analysis. A quality control sample was prepared by pooling equal amounts from all replicates. To ensure analytical consistency and data reliability, identical experimental conditions were applied to all samples. Lipid analysis was performed using ultra-high-performance liquid chromatography-mass spectrometry (UHPLC-MS) with a Q Exactive Plus high-resolution mass spectrometer (Thermo Scientific), coupled to an Ultimate 3000 UHPLC system (Thermo Scientific). Lipid identification and peak quantification, including retention time, m/z, and peak area, were processed using MS-DIAL software. The relative lipid abundances were determined by combining data from both positive and negative ion modes and used for subsequent statistical analysis.

### Malonyl-CoA and acetyl-CoA level detection

Cells were lysed and centrifuged (12,000 rpm, 10 min,4 °C), and the supernatants were aliquoted for immediate analysis. According to the manufacturer’s protocol (Jingmei Biotechnology Co., Ltd., Jiangsu, China), standards and 5-fold diluted samples were loaded in triplicate onto precoated plates (30 min, 37 °C), the wells were washed five times with TBST buffer, incubated with enzyme-linked reagents (30 min, 37 °C), and developed using chromogenic substrates (A/B, 10 min, 37 °C, in the dark). The reaction was terminated by adding 50 µL of termination solution to each well, and the absorbance was measured at 450 nm.

### scRNA-seq analysis

scRNA-seq analysis was performed as described previously [19]. Briefly, four publicly available single-cell RNA-seq datasets (GSE138709, GSE171899, GSE201425, GSE189903) were integrated using Seurat v4.0 with reciprocal PCA-based integration. Raw UMI counts were log-normalized (normalization.method = “LogNormalize”, scale.factor = 10,000) followed by variable feature selection (nfeatures = 2,000). Principal component analysis (PCA) was performed on scaled data (scale.factor = 10,000) using the top 30 principal components. Cell clustering was executed via graph-based Leiden algorithm at resolution 0.8, yielding seven major cellular lineages. Cholangiocyte subclustering was performed by extracting epithelial cells, followed by CopyKat v1.1.0 analysis for malignant/non-malignant stratification. Pathway enrichment analysis was conducted on malignant versus non-malignant cholangiocytes using AUCell and GSVA with KEGG metabolic pathway gene sets. Metabolic flux topology was reconstructed via scFEA (v1.1) using the COBRA-based model with default reaction networks [20]. Flux vectors were visualized in UMAP space following module completion analysis.

### Statistical analysis

Data were analyzed using GraphPad Prism 8.0. Two-group comparisons were assessed by unpaired T-test, while multi-group comparisons were evaluated via One-way ANOVA. Results are expressed as mean ± SEM. Statistical significance was defined as *P* < 0.05 (*), *P* < 0.01(**), and *P* < 0.001(***).

## Results

### WA significantly attenuates lipid accumulation in CCA

**Fig. 1:**
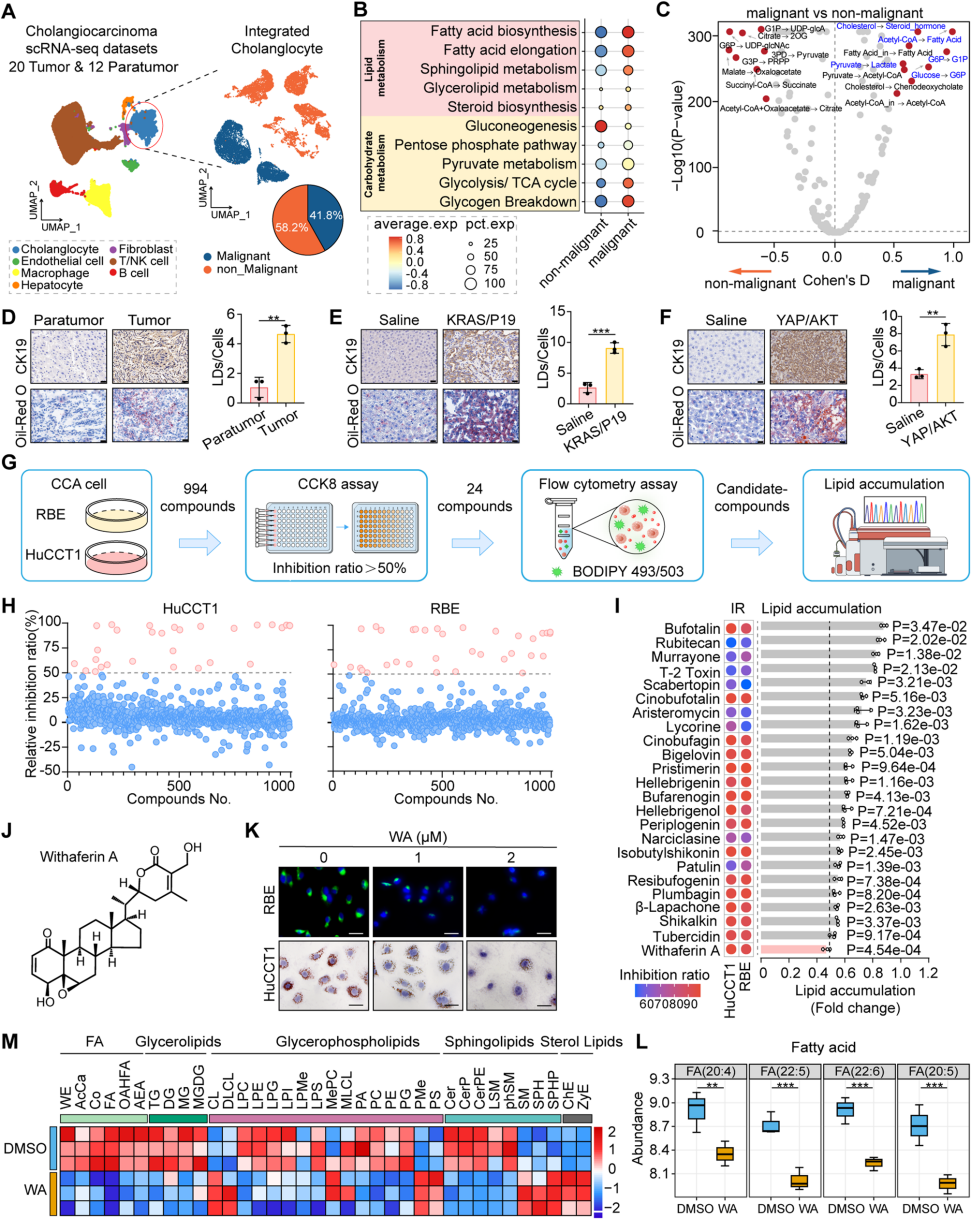
WA significantly attenuates lipid accumulation in CCA. **A** UMAP visualization of integrated scRNA-seq data (20 tumors, 12 adjacent tissues) showing seven major cell lineages, and malignant cholangiocyte identification by CopyKat analysis (dark blue: malignant, yellow: non-malignant). **B** Metabolic pathway enrichment in malignant versus non-malignant cholangiocytes. **C** Single-cell flux estimation analysis (scFEA) was performed to infer metabolic fluxes at single-cell resolution, revealing differential metabolite profiles and metabolic activities between malignant and non-malignant cholangiocytes. **D** CK19 IHC staining and oil red O staining to detect lipid accumulation in human CCA tissues, scale bar = 20 μm. **E** CK19 IHC staining and oil red O staining to detect lipid accumulation in the KRAS/P19 CCA model, scale bar = 20 μm. **F** CK19 IHC staining and oil red O staining to detect lipid accumulation in the YAP/AKT CCA model, scale bar = 20 μm. **G** The schematic for screening of anti-tumor inhibitors targeting lipid accumulation in a natural compound library containing 994 compounds. **H** The inhibitory rate of each inhibitor on HuCCT1 and RBE was detected by CCK8 assay. Red coloration indicated inhibitors that have achieved more than 50% inhibition. **I** BODIPY493/503 fluorescence staining and flow cytometry experiments were performed to detect lipid droplet content in HuCCT1 and RBE after treatment with each of the 24 compounds. **J** Molecular structure of WA. **K** This study employed a combination of BODIPY 493/503 fluorescence staining and Oil Red O staining to detect lipid droplet content in RBE and HuCCT1 cells 24 h after WA treatment. scale bar = 100 μm. **L**-**M** Lipids in WA treatment CCA cells were measured by LC-MS. The heatmap highlighted relative changes for each type of lipid metabolite with their individual average ion counts normalized to NTC. Data are representative of three independent experiments. scale bar = 100 μm. The data are represented as the mean ± SD of three independent experiments. * *P* < 0.05; ** *P* < 0.01; *** *P* < 0.001; ns, no significance

To investigate lipid metabolic dysregulation during CCA malignant progression, we integrated four independent single-cell RNA-seq datasets (GSE138709, GSE171899, GSE201425, GSE189903) comprising 20 tumors and 12 matched adjacent tissues. Following gene normalization and principal component analysis, graph-based clustering identified seven major cellular lineages. Cholangiocarcinoma epithelial cells were subsequently stratified into malignant and non-malignant subpopulations using the CopyKat algorithm for copy number variation inference (Fig. 1A). Enrichment analysis further uncovered pronounced carbohydrate metabolism pathways alterations specifically in malignant cholangiocytes, including glycogen breakdown and glycolysis/TCA cycle activity, concomitant with suppressed gluconeogenesis. Notably, lipid metabolic pathways (fatty acid biosynthesis, elongation, and sphingolipid metabolism) were coordinately upregulated (Fig. 1B). Single-cell flux estimation analysis (scFEA) further demonstrated concurrent metabolic shifts: accelerated glucose-to-G6P and G6P-to-G1P conversions occurred alongside lipogenic activation evidenced by acetyl-CoA-to-fatty acid and cholesterol-to-steroid hormone transitions, indicating synchronized glycogen degradation and lipid accumulation (Fig. 1C). This integrated omics approach reveals a coordinated reprogramming of glycogen degradation and lipogenesis in malignant cholangiocytes. Consistently, Histopathological analysis confirmed significant lipid deposition in malignant tissues relative to adjacent normal controls; a phenotype was recapitulated in vivo using two orthotopic CCA murine models (YAP/AKT vs. saline controls; KRAS/p19 vs. saline controls) (Fig. 1D-F, Supplementary Fig. 1A-B).

To identify pharmacological agents capable of targeting this metabolic vulnerability, we implemented a high-throughput screen of 994 bioactive compound library through a dual-phase strategy. Primary screening for anti-proliferative activity (Inhibition ratio > 50%) in HuCCT1 and RBE cells identified 29 and 34 hits respectively, yielded 24 overlapping candidates (Fig. 1G-H, Supplementary Table 1). Secondary screening via BODIPY 493/503 staining selected compounds with lipid-lowering efficacy and identified Withaferin A (WA) as the lead compound *P* = 4.54e-04), demonstrating dual suppression of CCA cell proliferation and lipid accumulation (Fig. 1I-K, Supplementary Fig. 1 C, Supplementary Fig. 2A-D). Complementary liquid chromatography–mass spectrometry (LC-MS) profiling of global lipid species upon WA treatment revealed significant reductions in multiple lipid classes, including fatty acid (FA, e.g., FA 20:4, 22:5, 22:6, 20:5), Glycerolipids (triglycerides, TG; diglycerides, DG), glycerophospholipids, and sphingolipids, consistent with scFEA predictions (Fig. 1L-M). These findings establish WA as a preferential modulator of oncogenic lipid metabolism in CCA.

### WA suppresses CCA tumorigenesis in vivo and in vitro

**Fig. 2:**
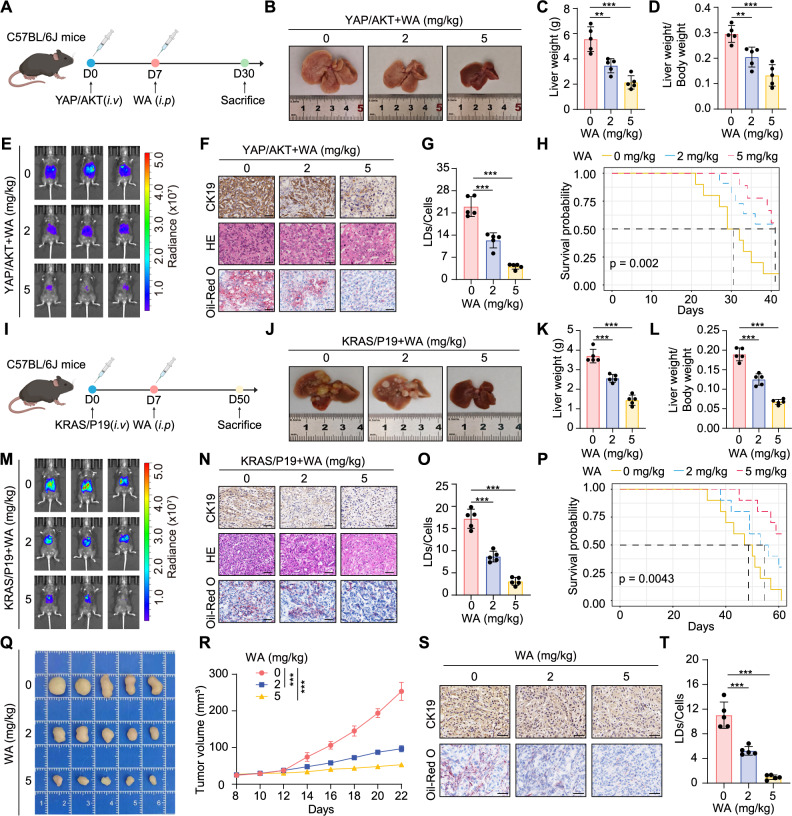
WA inhibits CCA tumorigenesis in vitro and in vivo. **A** Schematic diagram of CCA mouse model induced by YAP/AKT. Intraperitoneal injection with WA or saline after tumor formation. **B **Representative gross images of the YAP/AKT model after treatment. **C** Liver weights of YAP/AKT model after treatment. **D** The liver weight to body weight ratio (LBR) of YAP/AKT model. **E** Tumor burden of the YAP/AKT model detected by in vivo bioluminescence imaging (BLI). **F** Representative CK19 and oil red O staining images of the YAP/AKT model, scale bar = 50 μm. **G** Quantification of lipid droplets in the YAP/AKT model. **H** Survival curve of the YAP/AKT model after treatment with WA. **I** Schematic diagram of CCA mouse model induced by KRAS/P19. Intraperitoneal injection with WA or saline after tumor formation. **J** Representative gross images of the KRAS/P19 model after treatment. **K** Liver weights of KRAS/P19 model after treatment. **L** The LBR in KRAS/P19 model. **M** Tumor burden of the KRAS/P19 model detected by in vivo BLI. **N** Representative CK19 and oil red O staining images of the KRAS/P19 model, scale bar = 50 μm. **O** Quantification of lipid droplets in the KRAS/P19 model. **P** Survival curve of the KRAS/P19 model after treatment with WA. **Q** Representative image of the subcutaneous tumors from each group. **R** The tumor growth curves were generated by monitoring the tumor volume every other day. **S** Representative images of CK19 IHC staining and oil red O staining in the subcutaneous tumor model, scale bar = 50 μm. **T** The quantitative analysis of oil red O staining. The data are represented as the mean ± SD of three independent experiments. * *P* < 0.05; ** *P* < 0.01; *** *P* < 0.001; ns, no significance

To characterize the anti-tumor effects of WA, we conducted colony formation and transwell assays, revealing dose-dependent inhibition of CCA cell proliferation, migration, and invasion following WA treatment (Supplementary Fig. 3A-F). Flow cytometry analysis further revealed that WA treatment leads to a notable arrest in the G2/M phase in CCA cells (Supplementary Fig. 3G). Additionally, complementary western blot, flow cytometry, and TUNEL assays collectively confirmed WA-induced apoptotic cell death (Supplementary Fig. 4A-E). For in vivo evaluation, we employed the Sleeping Beauty (SB) transposon system to induce hepatic overexpression of oncogenic drivers myr-AKT/YAP^S127A^ (YAP/AKT) via hydrodynamic tail vein injection, followed by intraperitoneal WA administration (2 or 5 mg/kg) versus saline control (Fig. 2A). WA treatment significantly reduced tumor burden (Fig. 2B-E) and decreased cytokeratin 19-positive (CK19⁺) malignant cells, alongside diminished lipid droplet accumulation (Fig. 2F-G). Concurrently, the number of KI67-positive cells was reduced, and the process of apoptosis was increased in tumor tissues (Supplementary Fig. 5 A). Survival analysis showed that WA treatment significantly prolonged overall survival in tumor-bearing mice (Fig. 2H). Moreover, following WA treatment, no abnormalities observed in the body weight and major organs including brain, lungs, liver, spleen, and kidneys (Supplementary Fig. 5B-D). Consistent therapeutic efficacy was replicated in another primary CCA model established through KRAS^G12D^/P19 CRISPR (KRAS/P19) plasmids transfection (Fig. 2I-P, Supplementary Fig. 5E-H). Simultaneously, our tumor xenograft experiments results demonstrated diminished tumor burden, reduced tumor volume upon WA treatment, oil-Red staining results further confirmed a decrease in intratumoral lipid accumulation (Fig. 2Q-T). Collectively, these findings suggest WA as a dual-targeting agent that concurrently disrupts lipid reprogramming and tumor progression in CCA.

### WA directly targets ACC1 at Lys1338 to suppress lipid accumulation

**Fig. 3:**
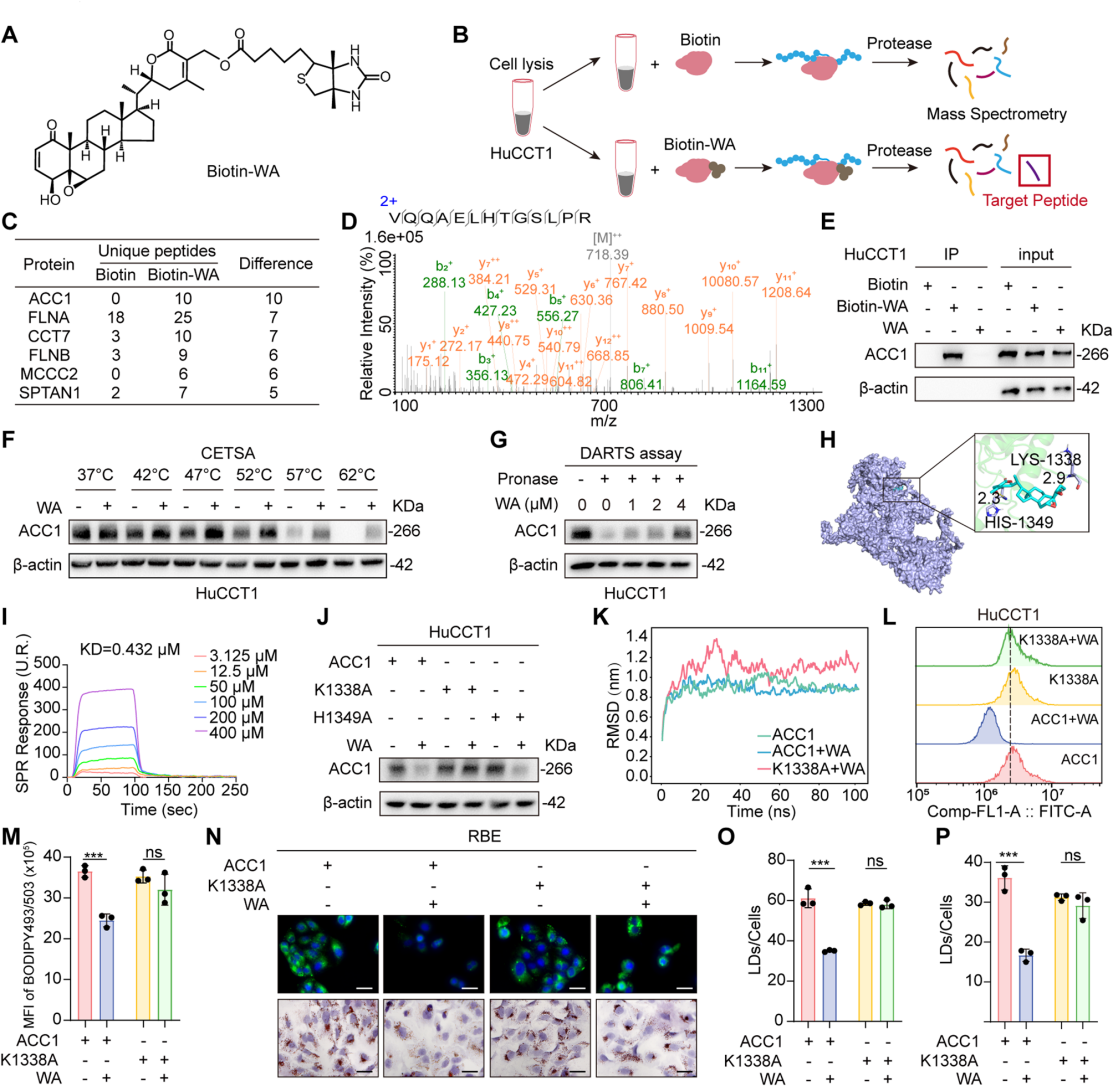
WA inhibits fatty acid synthesis by directly targeting ACC1-Lys1338. **A** Molecular structure of WA after biotin labeling. **B** Schematic diagram of pulldown-LC-MS/MS screening for WA binding protein. **C** The top 10 candidates of the Bio-WA interacting protein in terms of binding affinity. **D** MS/MS spectra of ACC1 peptides. **E** Pull down assay to detect the interaction of WA and ACC1 in HuCCT1. **F** CETSA assay to detect the interaction of WA and ACC1 in HuCCT1. **G** DARTS assay confirms that ACC1 is a direct target of WA in HuCCT1. **H** Schematic diagram of the docking of WA with the ACC1 protein. **I** SPR analysis to verify the binding of WA to ACC1. **J** HuCCT1 were treated with K1338 or H1349 mutant plasmids and treated with or without WA for 24 h. Western Blot was performed to detect the expression level of ACC1. **K** Line plots of RMSD values of ACC1 protein over time under conditions of WA treatment or transfection with K1338 plasmid. **L** HuCCT1 were treated with K1338 mutant plasmids and treated with or without WA for 24 h. BODIPY493/503 fluorescence staining and flow cytometry experiments were performed to detect the content of lipid droplets in the cells. **M** Quantitative analysis of lipid droplet content in HuCCT1. **N** BODIPY493/503 fluorescent staining and oil red O staining were used to detect changes in lipid droplet content of RBE transfected with the K1338 mutant plasmid after treatment with or without WA. scale bar = 100 μm. **O** Quantitative of BODIPY493/503 fluorescence staining. **P** Quantitative of oil red O staining. The data are represented as the mean ± SD of three independent experiments. * *P* < 0.05; ** *P* < 0.01; *** *P* < 0.001; ns, no significance

To elucidate the molecular basis of WA-mediated lipid homeostasis regulation in CCA, we synthesized a biotinylated WA derivative (Biotin-WA) for target identification (Fig. 3A-B, Supplementary Fig. 6A-C). Biotin-WA maintained potent inhibition of HuCCT1 cell viability, confirming bioactivity preservation post-conjugation (Supplementary Fig. 7 A). Liquid chromatography-tandem mass spectrometry (LC-MS/MS) profiling identified acetyl-CoA carboxylase 1 (ACC1), the rate-limiting enzyme in *de novo* fatty acid biosynthesis, as the principal binding target (Fig. 3C-D). Analysis of the Fu-icca database revealed a negative correlation between ACC1 mRNA expression levels and overall survival in CCA patients. (Supplementary Fig. 7B). Co-immunoprecipitation (Co-IP) assays validated direct interaction between Biotin-WA and ACC1 in CCA lysates (Fig. 3E). Cellular thermal shift assays (CETSA) demonstrated enhanced ACC1 thermostability (37–62 °C) upon WA binding (Fig. 3F, Supplementary Fig. 7 C), while drug affinity responsive target stability (DARTS) assays revealed concentration-dependent proteolytic susceptibility (Fig. 3G, Supplementary Fig. 7D), collectively confirming direct target engagement. Moreover, overexpression of ACC1 partially restored lipid synthesis in WA-treated cells (Supplementary Fig. 7E).

To identify potential binding sites of WA on ACC1, we performed molecular docking analyses, and the result identified a putative interaction between WA and the Lys1338 and His1349 residues within ACC1 structure (Fig. 3H). Surface plasmon resonance (SPR) analysis quantified a high-affinity WA–ACC1 binding, specifically within the 1200–1400 amino acid domain, with a dissociation constant (KD) of 0.432 µM (Fig. 3I). Notably, ACC1 K1338A mutation abrogated WA-induced ACC1 degradation, whereas H1349R substitution failed to rescue this phenotype (Fig. 3J, Supplementary Fig. 7 F). Molecular dynamics simulations further demonstrated WA-induced ACC1 structural stabilization that was abolished by K1338A mutation (Fig. 3K). Consistent with these findings, K1338A transfection reversed WA-mediated suppression of lipid accumulation (Fig. 3L-P, Supplementary Fig. 7G-H). Collectively, these results identify Lys1338 as the essential residue governing WA-ACC1 binding and subsequent disruption of lipid metabolism in CCA.

### WA downregulates ACC1 malonylation and protein levels

**Fig. 4:**
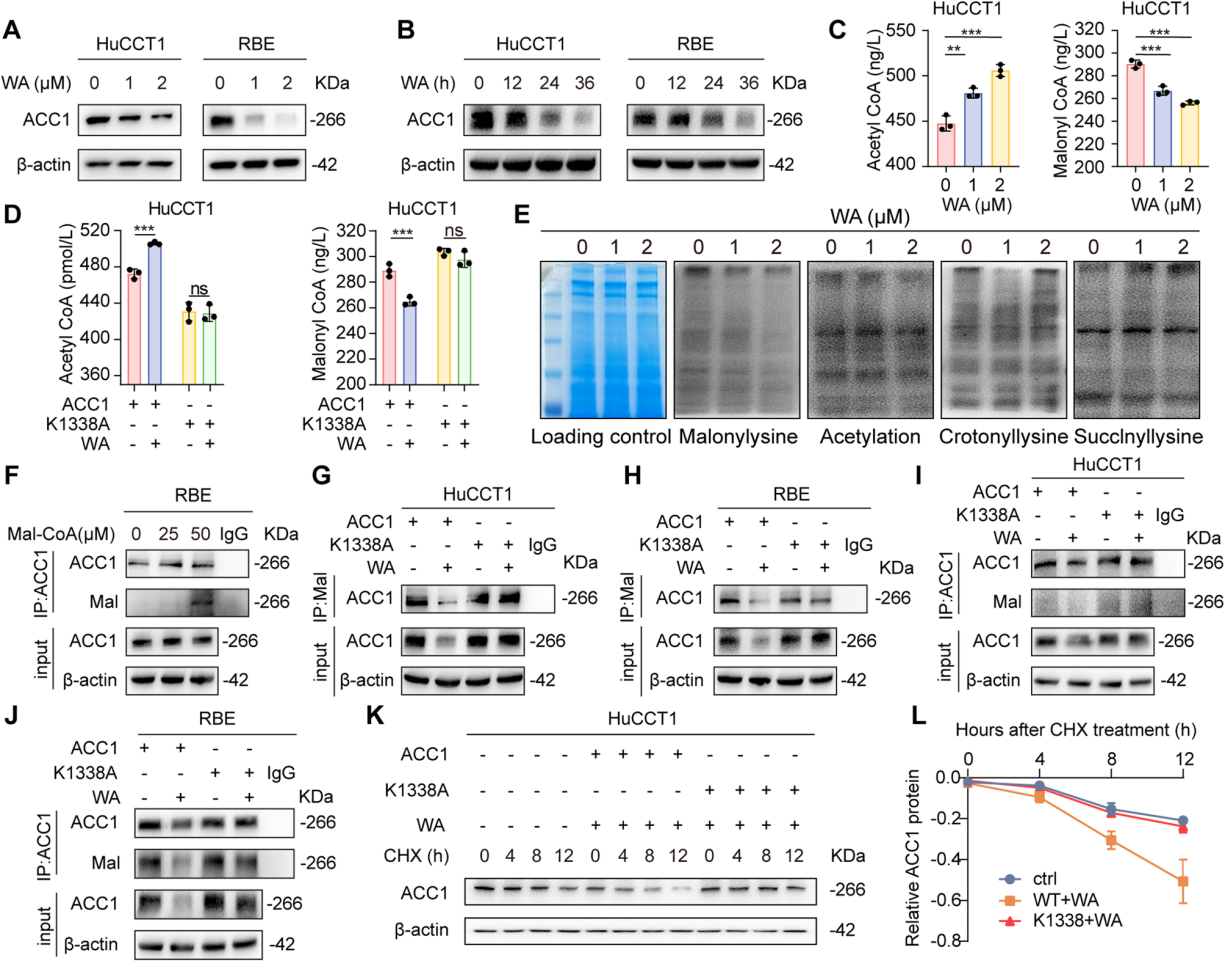
WA reduces ACC1 malonylation to regulate its protein stability. **A** Western blot detection of ACC1 protein levels in HuCCT1 and RBE cells after 24 h treatment with 0, 1, and 2 µM WA. **B** Western blot analysis of ACC1 protein levels in HuCCT1 and RBE cells after treatment with 1 µM WA at 0, 12, 24 and 36 h. **C** The detection of acetyl-coenzyme A and malonyl-coenzyme A in HuCCT1 treated with WA. **D** HuCCT1 was transfected with wild-type ACC1 or K1338 mutant plasmid, the levels of acetyl coenzyme A and malonyl coenzyme A were detected after WA treatment. **E **Western Blot detection of total protein malonylation, acetylation, crotonylation and succinylation levels in WA-treated HuCCT1 cells. **F** Detection of the ACC1 malonylation level after Malonyl coenzyme A treatment by Co-IP assay. **G**-**H** HuCCT1 and RBE were treated with K1338 mutant plasmids and treated with or without WA for 24 h. Co-IP assay was performed to detect the malonylation level of ACC1. **I**-**J** HuCCT1 and RBE were treated with K1338 mutant plasmids and treated with or without WA for 24 h. Co-IP assay was performed to detect the malonylation level of ACC1. **K**-**L** Western Blot detection of ACC1 protein levels (K) and related protein levels fold plots (L) after WA treatment of HuCCT1 for 24 h, followed by CHX treatment for 0, 4, 8, and 12 h. The data are represented as the mean ± SD of three independent experiments. * *P* < 0.05; ** *P* < 0.01; *** *P* < 0.001; ns, no significance

To elucidate the regulatory mechanism of WA on ACC1 expression, we systematically assessed its effects on ACC1 levels. Our initial results revealed that WA treatment significantly reduced ACC1 protein levels in a dose- and time-dependent manner, while qPCR confirmed no alteration in ACC1 mRNA expression, indicating post-translational regulation (Fig. 4A-B, Supplementary Fig. 8A-B). Given that ACC1 catalyzes acetyl-coenzyme A (CoA) to malonyl-CoA [21], we further measured intracellular levels of these metabolites following WA treatment, and the results demonstrated that WA significantly decreased malonyl-CoA levels while concomitantly increasing acetyl-CoA concentrations (Fig. 4C, Supplementary Fig. 8C-D). Notably, these metabolic shifts were abolished in cells expressing the ACC1-K1338A mutant (Fig. 4D, Supplementary Fig. 8E-F), establishing Lys1338 as essential for WA-mediated suppression of ACC1 enzymatic activity and metabolic function.

Emerging evidence indicates that intermediate metabolites serve as donors for lysine-directed protein post-translational modifications (PTMs) (e.g., acetyl-CoA for acetylation; malonyl-CoA for malonylation) [22, 23]. To investigate PTMs involvement in WA-alleviated lipid deposition, the cellular level of different types of lysine modifications was measured. Global malonylation level was significantly reduced upon WA treatment, with no obvious changes in total protein acetylation, crotonylation and succinylation (Fig. 4E). It is worth noting that elevated ACC1 malonylation and stability have been reported in high-fat diet (HFD)-induced hepatic steatosis [24]. Our subsequent Co-IP experiments in RBE confirmed that exogenous malonyl-CoA enhanced ACC1 malonylation (Fig. 4F). Furthermore, ACC1 malonylation level decreased in tumor tissues from WA-treated mice (Supplementary Fig. 9 A). Concurrently, the addition of exogenous malonyl-CoA abolished WA-induced degradation of ACC1 and partially restored its malonylation level (Supplementary Fig. 9B). Further experiments revealed that WA treatment substantially suppressed ACC1 malonylation, and this suppression was abrogated by transfection with the K1338A mutant (Fig. 4G-J). Protein stability assays demonstrated that exogenous malonyl-CoA and K1338A mutation markedly attenuated WA-induced ACC1 degradation (Fig. 4K-L and Supplementary Fig. 9 C). Collectively, these results establish that Lys1338-dependent suppression of ACC1 malonylation mediates WA-induced regulation of ACC1 protein stability.

### WA accelerates the autophagic degradation of ACC1

**Fig. 5:**
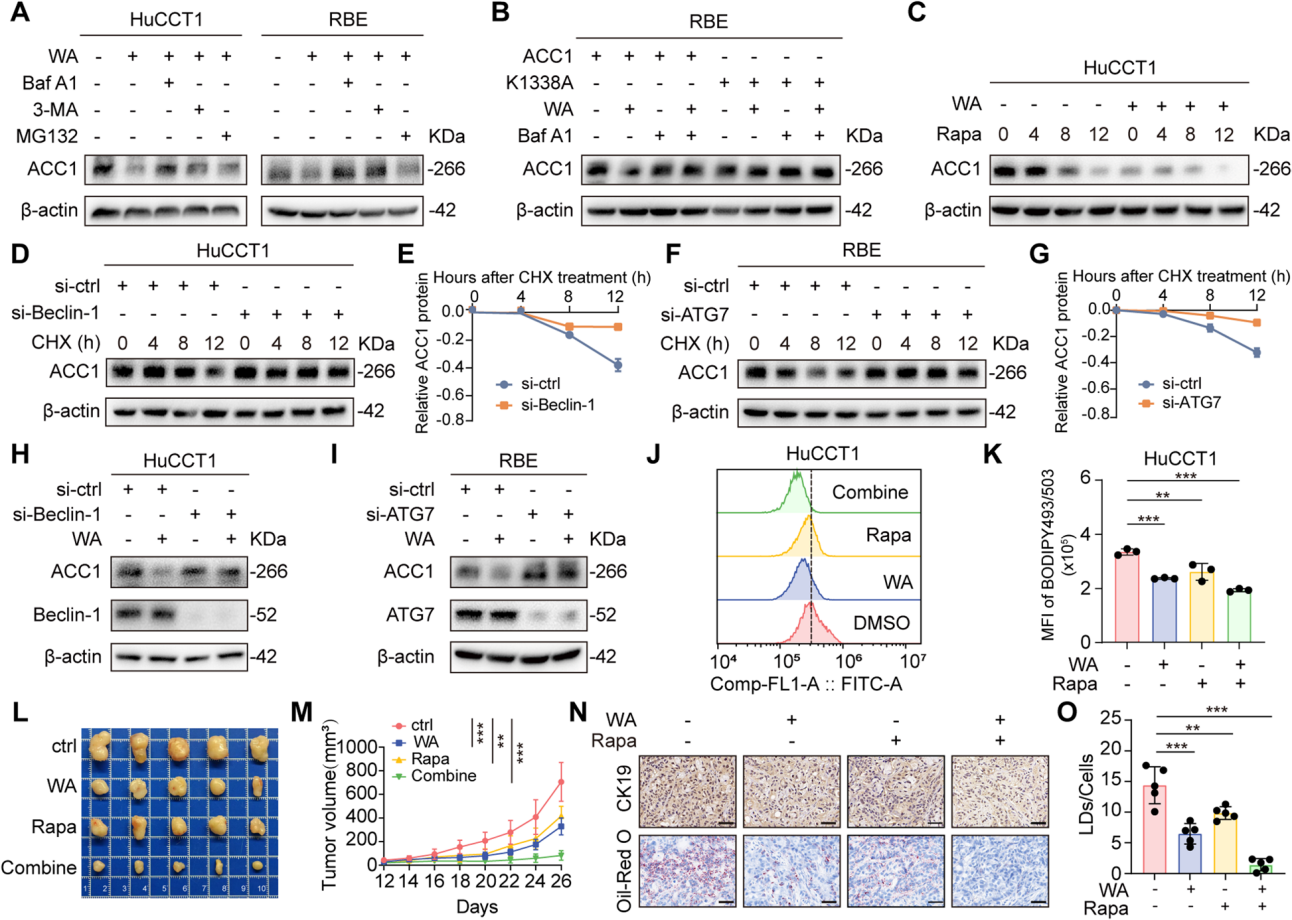
WA accelerates the autophagic degradation of ACC1. **A** Western Blot detection of ACC1 protein levels in HuCCT1 and RBE after treatment with WA or the proteasome inhibitor MG132 or the autophagy inhibitors Baf A1, 3-MA. **B** RBE transfected with wild-type ACC1 or the K1338 mutant plasmid were treated with WA or Baf A1, and Western Blot was used to detect the protein level of ACC1. **C** HuCCT1 were treated with WA for 24 h followed by rapamycin for 0, 4, 8, or 12 h. Western Blot was used to detect the protein level of ACC1 in HuCCT1 and RBE. **D**-**E** Changes in the expression levels of ACC1 protein in HuCCT1 of control and Beclin-1 knockdown groups after CHX (100 µg/mL) treatment at different time points (D) and Line charts of relevant statistics(E). **F**-**G** Changes in the expression levels of ACC1 protein in RBE of control and ATG7 knockdown groups after CHX (100 µg/mL) treatment at different time points (F) and Line charts of relevant statistics(G). **H** HuCCT1 before and after WA treatment to knock down Beclin-1, and protein levels of ACC1 and Beclin-1 were detected in the cells by Western Blot. **I** RBE before and after WA treatment to knock down ATG7, and protein levels of ACC1 and ATG7 were detected in the cells by Western Blot. **J** HuCCT1 were treated with WA or rapamycin, and the lipid droplet content in the cells was detected and quantified by BODIPY493/503 fluorescent staining and flow cytometry experiments. **K** Quantitative analysis of lipid droplet content. **L** Representative diagrams of subcutaneous tumors in each group. **M** Tumor growth graphs generated by monitoring tumor volume every other day. **N** Representative images of immunohistochemical CK19 and oil red O staining in the model of WA versus rapamycin treatment, scale bar = 50 μm. **O** Quantitative analysis of oil red O staining. The data are represented as the mean ± SD of three independent experiments. * *P* < 0.05; ** *P* < 0.01; *** *P* < 0.001; ns, no significance

To figure out the predominant degradation pathway responsible for WA-induced ACC1 degradation, we evaluated the effects of inhibitors targeting proteasomal and autophagy-lysosomal systems. Notably, our results showed that ACC1 degradation was effectively blocked by autophagy-lysosomal inhibitors bafilomycin A1 (Baf A1) and 3-Methyladenine (3-MA), whereas the proteasome inhibitor MG132 exhibited no such effect, indicating that WA promoted ACC1 degradation primarily through the autophagy-lysosome pathway (Fig. 5A). Furthermore, transfection with the ACC1-K1338A mutant attenuated ACC1 degradation to a similar extent as Baf A1 treatment, reinforcing the involvement of autophagy in WA-mediated regulation (Fig. 5B). We next investigated whether WA cooperates with canonical autophagy inducers to promote ACC1 degradation. Treatment with rapamycin (Rapa), a known autophagy activator, led to a significant reduction in ACC1 protein levels, an effect that was further enhanced by WA co-treatment (Fig. 5C). To establish a direct mechanistic link to autophagy machinery, we assessed ACC1 levels following genetic ablation of autophagy regulators. ACC1 degradation was rescued in ATG7- or Beclin1-deficient cells (Fig. 5D-G), and critically, WA-induced ACC1 degradation was substantially abrogated in both ATG7- and Beclin1-knockdown models (Fig. 5H-I). Taken together, these data indicate that WA treatment triggers autophagic degradation of ACC1.

To assess the functional impact of ACC1 degradation, we quantified lipid accumulation using BODIPY 493/503 staining. The results showed that combined treatment with WA and Rapa significantly reduced lipid accumulation in CCA cells compared to treated with WA or Rapa alone, which was further corroborated by oil Red O staining (Fig. 5J-K, Supplementary Fig. 10A-D). In vivo analyses confirmed that mice receiving combination therapy exhibited significantly attenuated tumor burden, decreased tumor volume, and diminished lipid droplet accumulation relative to single-agent treatment (Fig. 5L-O). Collectively, these data demonstrate that WA-mediated autophagic degradation of ACC1 substantially impairs lipid storage capacity, thereby contributing to its antitumor efficacy in CCA models.

### SQSTM1/p62 is required for WA-induced ACC1 degradation

**Fig. 6:**
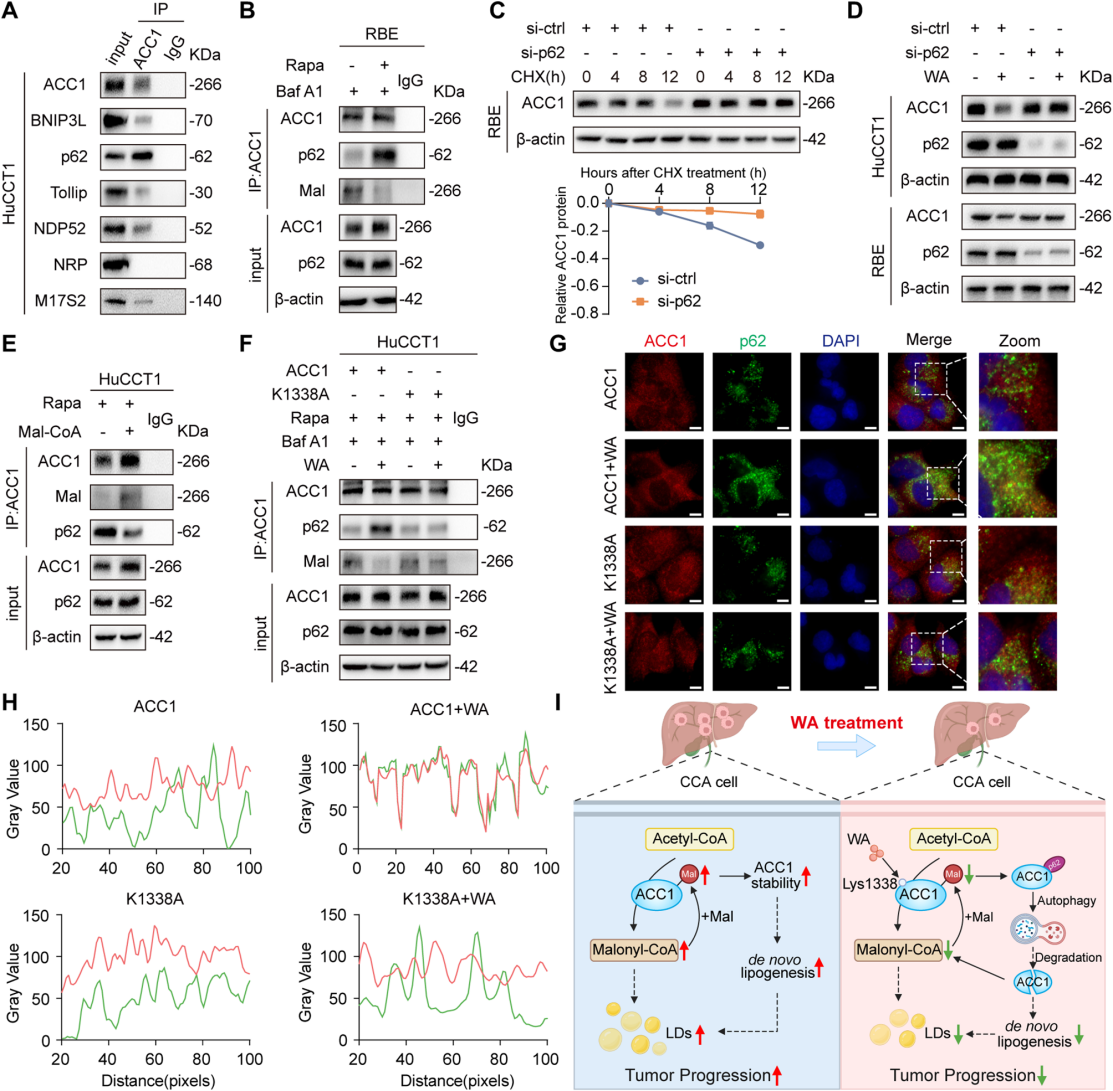
Cargo receptor SQSTM1/p62 is required for WA-induced ACC1 degradation. **A** Co-IP assay to detect the interaction of ACC1 with the autophagy proteins BNP3L, p62, Tollip, NDP52, NRP, and M17S2 in WA-treated HuCCT1. **B** Co-IP assay to detect the malonylation level of ACC1 and its interaction with p62 in RBE after rapamycin and Baf A1 treatment. **C** Changes in the expression levels of ACC1 protein in RBE of control and p62 knockdown groups after CHX (100 µg/mL) treatment at different time points (0–12 h) and line charts of relevant statistics. **D** Western Blot detection of protein levels of ACC1 and p62 in cells after treatment of HuCCT1 and RBE knocked down for p62 with WA. **E** Co-IP assay to detect the malonylation level of ACC1 and its interaction with p62 in HuCCT1 treated with rapamycin or malonyl coenzyme A. **F** HuCCT1 transfected with wild-type ACC1 or K1338 mutant plasmid were treated with rapamycin and Baf A1, then treated with WA or DMSO for 24 h, and Co-IP assay was performed to detect the level of malonylation of ACC1 and its interaction with p62 in cells. **G** HuCCT1 transfected with wild-type ACC1 or K1338 mutant plasmid were treated with rapamycin and Baf A1, then treated with WA or DMSO for 24 h, and immunofluorescence co-localization was used to analyze the interaction of ACC1 (red) with p62 (green), and DAPI (blue) labeled nuclei, scale bar = 20 μm. **H** Quantitative analysis of co-localization. **I** By binding to the 1338 site of ACC1 and inhibiting its malonylation modification, WA enhances the interaction between ACC1 and the autophagy receptor protein p62. This, in turn, promotes the degradation of ACC1 via the autophagy-lysosome pathway. Consequently, WA interferes with the lipid homeostasis of CCA cells, thereby inhibiting their malignant progression. The data are represented as the mean ± SD of three independent experiments. * *P* < 0.05; ** *P* < 0.01; *** *P* < 0.001; ns, no significance

During the autophagy process, cargo receptors mediate substrate delivery to autophagosomes for degradation [25]. To identify the specific cargo receptor facilitating ACC1 autophagic degradation, we screened a panel of established receptors, including BNIP3L, p62, Tollip, NDP52, NRP, and NBR1. Co-IP assays revealed that ACC1 predominantly interacts with SQSTM1/p62, with negligible binding to other candidates (Fig. 6A). Moreover, rapamycin-induced autophagy robustly enhanced the interaction of ACC1 and SQSTM1/p62 (Fig. 6B). Cycloheximide (CHX) chase assays further indicated that SQSTM1/p62 depletion abrogated ACC1 degradation (Fig. 6C), indicating SQSTM1/p62 is essential for autophagic clearance of ACC1.

To validate the necessity of SQSTM1/p62 in WA-induced ACC1 degradation, we monitored the protein level of ACC1 in cells following SQSTM1/p62 knockdown. WA treatment reduced ACC1 levels in control cells but failed to induce degradation in SQSTM1-deficient cells (Fig. 6D). We also found that malonylation of ACC1 by exogenous malonyl-CoA supplementation restricted the interaction between ACC1 and SQSTM1/p62 (Fig. 6E). Conversely, WA-mediated suppression of ACC1 malonylation strengthened this interaction, an effect attenuated by the ACC1-K1338A mutation (Fig. 6F). Confocal microscopy corroborated these findings, demonstrating enhanced ACC1–SQSTM1/p62 colocalization upon WA exposure, which was markedly reduced in K1338A-mutant cells (Fig. 6G-H). Collectively, our results demonstrate that inhibition of ACC1 malonylation by WA treatment increase the interaction between ACC1 and SQSTM1/p62, thereby facilitating ACC1 delivery to the autophagosome for selective degradation (Fig. 6I).

### WA potentiates gemcitabine efficacy in CCA

**Fig. 7:**
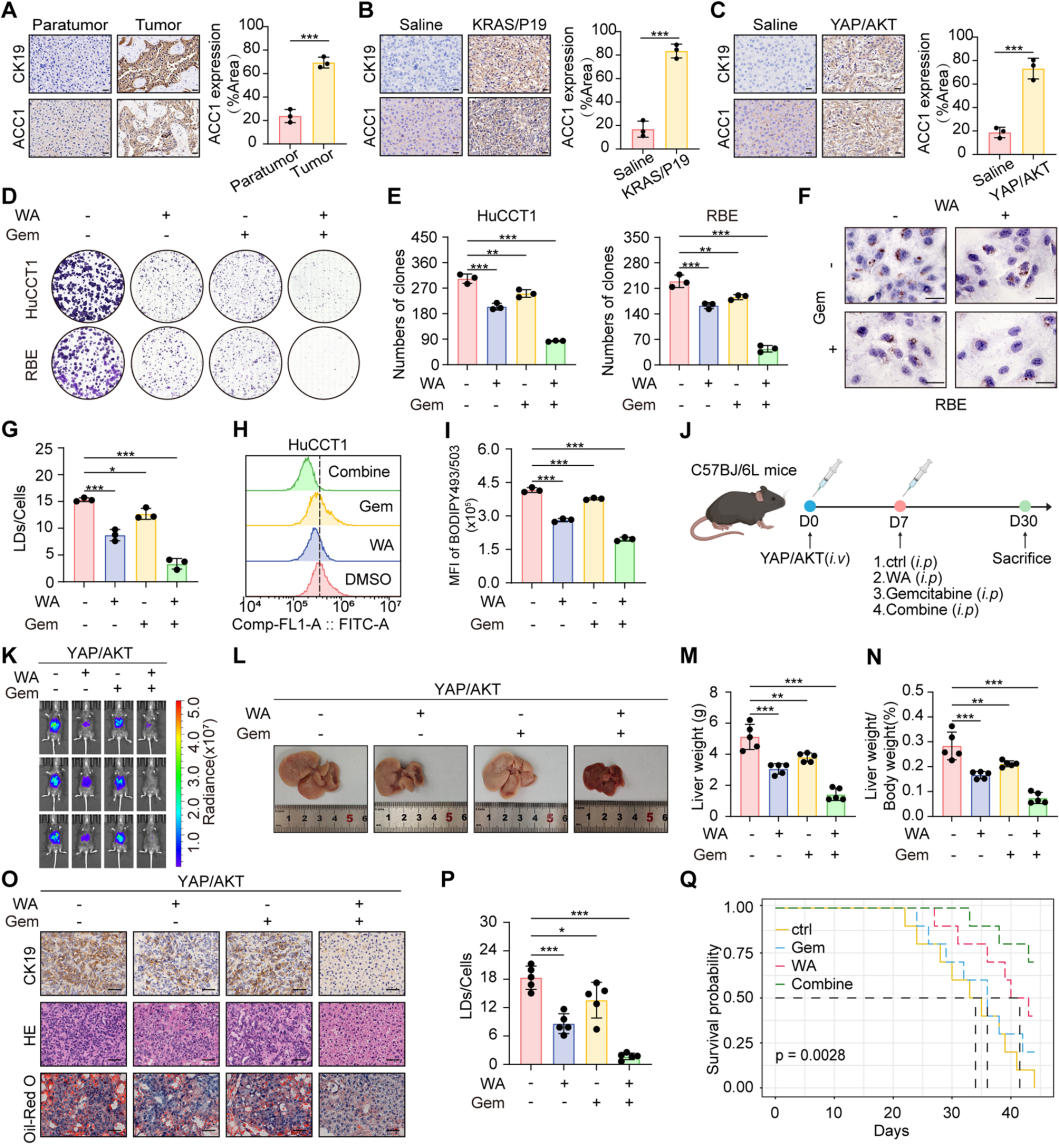
WA enhances the anticancer effect of gemcitabine. **A** Immunohistochemical staining was used to detect ACC1 levels in human CCA tissues, scale bar = 20 μm. **B** Immunohistochemical staining to detect ACC1 levels in CCA tissues of the KRAS/P19 model, scale bar = 20 μm. **C** Immunohistochemical staining to detect ACC1 levels in CCA tissues of the YAP/AKT model, scale bar = 20 μm. **D** Clone formation assay to verify the effect of WA or gemcitabine treatment on the proliferative capacity of HuCCT1 and RBE. **E** Quantitative analysis of clone formation experiments. **F** RBE were treated with WA or gemcitabine, and oil red O staining assay was performed to detect the lipid droplet content in the cells, scale bar = 100 μm. **G** Quantitative analysis of oil red O staining experiments. **H** HuCCT1 were treated with WA or gemcitabine, and lipid droplet content in the cells was detected by BODIPY493/503 fluorescent staining and flow cytometry experiments. **I** Quantitative analysis of BODIPY493/503 fluorescent staining. **J** Schematic diagram of CCA mouse model induced by YAP/AKT. Intraperitoneal injection with WA or gemcitabine after tumor formation. **K** In vivo BLI to detect tumor burden in YAP/AKT model after treatment with WA or gemcitabine. **L** Representative gross images of the YAP/AKT model after WA or gemcitabine treatment. **M** Liver weights of mice in the YAP/AKT model. **N** The LBR in YAP/AKT model. **O** Representative images of HE, immunohistochemical CK19, and oil red O staining. scale bar = 50 μm. **P** Statistical plot of lipid droplet content in mouse tissues. **Q** Survival curves for the YAP/AKT model. The data are represented as the mean ± SD of three independent experiments. * *P* < 0.05; ** *P* < 0.01; *** *P* < 0.001; ns, no significance

To evaluate the clinical relevance of ACC1, we performed immunohistochemical analysis on surgically resected human CCA specimens paired with adjacent non-tumorous tissues. Quantitative assessment revealed significantly elevated ACC1 protein levels in tumor tissues (Fig. 7A). Similar results were observed in YAP/AKT and KRAS/P19 mouse models confirming the clinical significance of targeting ACC1 (Fig. 7B-C). The gemcitabine (GEM)-based regimen remains the cornerstone of first-line chemotherapy, yet its efficacy is frequently limited by intrinsic or acquired resistance. Recent integration of targeted therapies with GEM aims to enhance synergistic antitumor effects and improve clinical outcomes [26–28]. Our study investigated whether WA could augment the effects of GEM in CCA. in vivo clonogenic assays revealed synergistic suppression of proliferative capacity in combinatorial WA/GEM treatment versus monotherapies (Fig. 7D-E). Critically, combinatorial therapy substantially attenuated neutral lipid accumulation compared to monotherapies (Fig. 7F-I). In a spontaneous CCA mouse model (Fig. 7J), bioluminescence imaging demonstrated profound tumor regression with combination treatment (Fig. 7K). Mice receiving combination therapy exhibited reduced tumor burden, diminished tumor volume and normalized liver weight coefficient relative to monotherapy groups. Histopathological analyses further confirmed attenuated CK19 immunoreactivity and lipid droplet deposition in combinatorial cohorts (Fig. 7L-P). Impressively, survival analyses demonstrated that WA/GEM co-administration significantly extended overall survival in tumor-bearing mice (Fig. 7Q). Collectively, these data underscore the potential of WA to synergize with conventional chemotherapy, offering a promising combinatorial strategy for improving patient outcomes against this aggressive malignancy.

## Discussion

CCA represents a formidable therapeutic challenge due to its intricate anatomical constraints, profound metabolic plasticity, and persistent chemoresistance [29, 30]. Dysregulated lipid metabolism sustains tumor progression by providing energy, biomembrane components, and signaling molecules essential for oncogenesis, positioning it as a promising therapeutic strategy [31–33]. In this study, we established WA as a novel inhibitor of lipid metabolism in CCA through direct targeting of ACC1. Furthermore, we revealed an unprecedented regulatory axis involving malonylation-dependent autophagic degradation. These findings not only provide mechanistic insights but also offer therapeutic promise for this challenging malignancy.

Cancer cells exhibit a heightened reliance on *de novo* lipogenesis, even in the presence of abundant extracellular lipids, with key enzymes such as ACLY, ACC1, and FASN identified as promising therapeutic targets [34–36]. The pervasive accumulation of lipid droplets in human CCA tissues and murine models highlights the dysregulation of lipid metabolism as a fundamental hallmark of cholangiocarcinogenesis. This phenotype is consistent with the crucial role of lipogenesis in supplying membranes, signaling molecules, and energy for rapidly proliferating tumors. While therapeutic strategies such as FASN inhibition or ACLY/SCD1 synthetic lethality have shown promise in other cancers [37, 38], the lipid metabolic dependencies specific to CCA remain largely unexplored. Our high-throughput screening identified WA as a potent dual inhibitor of both proliferation and lipid accumulation, providing direct pharmacological evidence that disrupting lipid homeostasis is detrimental to CCA survival. The significant reduction in lipid droplets observed in vivo following WA treatment, which correlates with suppressed tumor growth, further substantiates lipid metabolism as a therapeutically actionable vulnerability in CCA. This positions WA uniquely among agents that solely target proliferation or apoptosis, offering a mechanism to undermine a fundamental metabolic support system.

Withaferin A, a steroidal lactone extracted from Solanum nigrum, has garnered significant attention due to its potent anticancer properties. Previous studies have shown that Withaferin A can effectively induce apoptosis in cancer cells, arrest cell cycle progression [39], and modulate critical signaling pathways such as NF-κB [40], ultimately inhibiting tumor growth and metastasis. These findings suggest its potential as a novel therapeutic agent in cancer treatment. Notably, recent research has highlighted its additional role in stabilizing IDH1 and enhancing mitochondrial function, which may suppress tumorigenesis by mitigating cellular metabolic stress [41]. Furthermore, Withaferin A has demonstrated promising therapeutic potential in the treatment of ischemic retinal diseases, expanding its scope beyond oncology. These multifaceted effects underline the importance of further investigation into Withaferin A’s molecular mechanisms and its clinical applicability in various disease contexts. In this study, we established WA as a novel inhibitor of lipid metabolism in CCA through direct targeting of ACC1. Furthermore, we revealed an unprecedented regulatory axis involving malonylation-dependent autophagic degradation. These findings not only provide mechanistic insights but also offer therapeutic promise for this challenging malignancy. Our discovery that WA-induced malonyl-CoA depletion suppresses ACC1 auto-malonylation represented a major conceptual advance. While lysine malonylation is an emerging post-translational modification (PTM), its functional role in regulating enzyme stability has been minimally explored in malignancy [42]. Herein, we demonstrated that ACC1 malonylation, enhanced by exogenous malonyl-CoA, functions as a stabilizing signal that protects ACC1 from degradation. WA-induced depletion of malonyl-CoA effectively removed this protective PTM, thereby exposing ACC1 to degradation. This finding established ACC1 malonylation as a direct metabolic sensor, linking intracellular malonyl-CoA concentration to its own protein stability through an auto-regulatory loop. This mechanism elucidated the WA-induced post-translational downregulation of ACC1 protein without affecting mRNA levels. The dependency on Lys1338 indicated that this residue is not only critical for WA binding but also likely serves as a key malonylation site, positioning WA binding and malonylation status as interdependent regulators of ACC1 fate. This novel metabolite-PTM-stability axis significantly broadened our understanding of ACC1 regulation beyond the canonical kinase-regulated PTMs.

Autophagy is a highly conserved process mediating lysosomal degradation and recycling of cellular contents, achieved through selective cargo receptors such as SQSTM1/p62, OPTN, and NDP52 [43]. These receptors recognize substrates via ubiquitin-binding domains or other recognition signals, tethering cargo to LC3-decorated autophagosomal membranes [44]. Specifically, cargo receptors facilitate substrate recognition by bridging ATG family proteins to ubiquitin chains or alternative selective signals on cargo molecules [45]. In this study, we demonstrated that WA induces selective autophagic degradation of ACC1, representing a paradigm shift in understanding metabolite-directed protein turnover. Genetic validation confirmed absolute dependence on core autophagy machinery, as ablation of ATG7 or Beclin1 completely rescued ACC1 protein levels. Crucially, among multiple autophagy receptors screened, SQSTM1/p62 emerged as the exclusive molecular bridge between demalonylated ACC1 and the autophagy apparatus. The pivotal breakthrough came from elucidating how malonylation status governs receptor engagement: malonylation acted as a shield for preventing SQSTM1/p62 binding, while WA-induced demalonylation exposed an interaction interface. This revealed a novel “demalonylation-tag” mechanism distinct from classical ubiquitin-mediated recognition. This model was further substantiated by the observation that the K1338A mutant, which resisted both demalonylation and SQSTM1/p62 binding, consequently evading degradation. Collectively, these findings established a metabolite-regulated hierarchical cascade wherein WA binding-initiated malonyl-CoA depletion, triggering diminished ACC1 malonylation that licenses SQSTM1/p62 recognition and subsequent autophagic degradation. This constitutes the first demonstration of malonylation serving as a reversible degradation switch directing selective autophagy.

## Conclusion

In summary, this study demonstrated that inhibition of ACC1 malonylation by WA treatment increase the interaction between ACC1 and SQSTM1/p62, thereby facilitating ACC1 delivery to the autophagosome for selective degradation, thereby disrupting lipid accumulation and malignant progression. Notably, we established ACC1 malonylation as a clinically actionable target for lipid metabolism-driven malignancies, offering a mechanistic foundation for novel combinatorial regimens targeting the malonylation-autophagy axis to overcome therapeutic resistance in cholangiocarcinoma.

## Supplementary Information

Supplementary Material 1.

Supplementary Material 2.

Supplementary Material 3.

## Data Availability

No datasets were generated or analysed during the current study.

## Abbreviations

CCA: Cholangiocarcinoma
WA: Withaferin A
ACC1: Acetyl-CoA carboxylase 1
PYGB: Glycogen Phosphorylase B
PPP: Pentose phosphate pathway
FASN: Fatty acid synthase
ACLY: ATP-citrate lyase
SCD1: Stearoyl-CoA desaturase 1
scRNA-seq: Single-cell RNA sequencing
FBS: Fetal bovine serum
SB: Sleeping Beauty transposase
CCK-8: Cell Counting Kit-8
IF: Immunofluorescence
SPR: Surface plasmon resonance
DARTS: Drug affinity responsive target stability
CETSA: Cellular thermal shift assay
scFEA: Single-cell flux estimation analysis
LC-MS: Liquid chromatography–mass spectrometry
FA: Fatty acid
CK19: Cytokeratin 19
BLI: Bioluminescence imaging
Biotin-WA: Biotinylated WA derivative
Co-IP: Co-immunoprecipitation
CoA: Coenzyme A
PTMs: Post-translational modifications
CHX: Cycloheximide
Baf A1: Bafilomycin A1
3-MA: 3-Methyladenine
Rapa: Rapamycin
GEM: Gemcitabine
