# Supplementary material for: Pharmacological induction of acetyl-CoA carboxylase 1 autophagic degradation attenuates lipid accumulation and cholangiocarcinoma progression
Source: J Exp Clin Cancer Res. 2025 Nov 25;44:310. doi: 10.1186/s13046-025-03564-8 (PMC12645744; doi:10.1186/s13046-025-03564-8)
Supplement: Supplementary file 1 — Supplementary Material 1. [file 13046_2025_3564_MOESM1_ESM.docx]

**Supplementary Table 1. The 24 overlapped compounds suppressed proliferation in HuCCT1 and RBE cells**

| **CAS NO.** | **Name** | **Formula** | **Inhibition Ratio (%)** | |
| --- | --- | --- | --- | --- |
|  |  |  | **Hucct1** | **RBE** |
| 1258-84-0 | Pristimerin | C_30_H_40_O_4_ | 98.21 | 98.77 |
| 508-79-2 | Hellebrigenol | C_24_H_34_O_6_ | 98.03 | 86.60 |
| 471-95-4 | Bufotalin | C_26_H_36_O_6_ | 97.81 | 89.45 |
| 470-37-1 | Cinobufagin | C_26_H_34_O_6_ | 97.71 | 92.44 |
| 4707-32-8 | β-Lapachone | C_15_H_14_O_3_ | 97.45 | 91.95 |
| 1108-68-5 | Cinobufotalin | C_26_H_34_O_7_ | 97.29 | 95.72 |
| 52438-12-7 | Isobutylshikonin | C_20_H_22_O_6_ | 97.25 | 96.56 |
| 5119-48-2 | Withaferin A | C_28_H_38_O_6_ | 97.15 | 93.28 |
| 17008-65-0 | Bufarenogin | C_24_H_32_O_6_ | 96.55 | 97.85 |
| 54952-43-1 | (Rac)-Shikonin | C_16_H_16_O_5_ | 95.53 | 96.67 |
| 481-42-5 | Plumbagin | C_11_H_8_O_3_ | 95.19 | 92.24 |
| 3668-14-2 | Bigelovin | C_17_H_20_O_5_ | 94.52 | 94.14 |
| 69-33-0 | Tubercidin | C_11_H_14_N_4_O_4_ | 93.07 | 91.48 |
| 465-39-4 | Resibufogenin | C_24_H_32_O4 | 92.87 | 95.05 |
| 465-90-7 | Hellebrigenin | C_24_H_32_O_6_ | 90.76 | 96.24 |
| 514-39-6 | Periplogenin | C_23_H_34_O_5_ | 89.81 | 95.24 |
| 476-28-8 | Lycorine | C_16_H_17_NO_4_ | 72.80 | 54.11 |
| 29477-83-6 | Narciclasine | C_14_H_13_NO_7_ | 71.96 | 66.17 |
| 19186-33-5 | Aristeromycin | C_11_H_15_N_5_O_3_ | 63.13 | 56.32 |
| 21259-20-1 | T-2 Toxin | C_24_H_34_O_9_ | 61.47 | 62.61 |
| 149-29-1 | Patulin | C_7_H_6_O_4_ | 60.10 | 73.45 |
| 19668-69-0 | Murrayone | C_15_H_14_O_4_ | 58.56 | 71.26 |
| 185213-52-9 | Scabertopin | C_20_H_22_O_6_ | 55.55 | 51.68 |
| 91421-42-0 | Rubitecan | C_20_H_15_N_3_O_6_ | 51.88 | 57.20 |
