# Supplementary material for: Pharmacological induction of acetyl-CoA carboxylase 1 autophagic degradation attenuates lipid accumulation and cholangiocarcinoma progression
Source: J Exp Clin Cancer Res. 2025 Nov 25;44:310. doi: 10.1186/s13046-025-03564-8 (PMC12645744; doi:10.1186/s13046-025-03564-8)
Supplement: Supplementary file 2 — Supplementary Material 2. [file 13046_2025_3564_MOESM2_ESM.docx]

**Supplementary Materials for**

**Pharmacological Induction of Acetyl-CoA Carboxylase 1 Autophagic Degradation Attenuates Lipid Accumulation and Cholangiocarcinoma Progression**

Yani Pan, Nannan Zhang, Xueni Fu, Xinyu Wang, Yichun Ma, Qi Chen, Yue Zhou, Hongwen Liu, Yun Zhu, Lei Xu, Qiang Wang, Dongyin Chen*, Zhangding Wang*, Lei Wang*

*Correspondence: [leiwang9631@nju.edu.cn](mailto:leiwang9631@nju.edu.cn) (L.W); zdwang@ahmu.edu.cn (Z.W); [chendongyin@njmu.edu.cn](mailto:chendongyin@njmu.edu.cn) (D.C)

This file includes:

Supplementary Figures

Supplementary Figure Legends


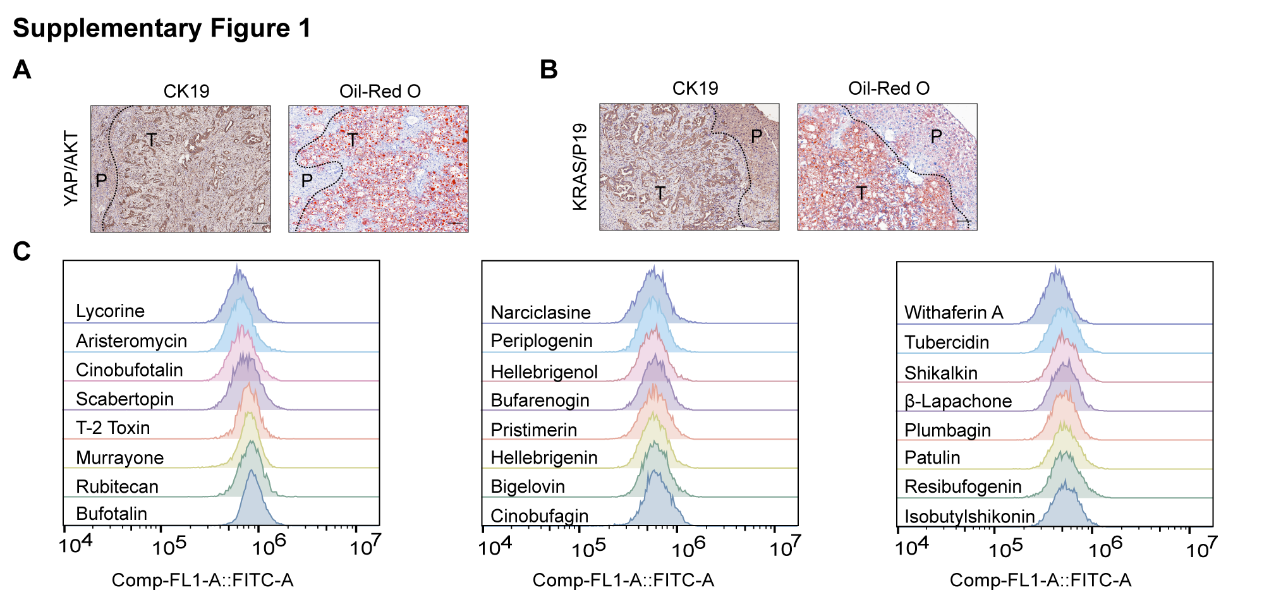


**Supplementary Figure 1.** (A) Low-magnification images of CK19 staining and Oil-Red O staining to detect the effect of WA treatment in YAP/AKT model, scale bar = 20 μm. (B) Low-magnification images of CK19 staining and Oil-Red O staining to detect the effect of WA treatment in KRAS/P19 model, scale bar = 20 μm. (C) Flow cytometry analysis of lipid droplet content in HuCCT1 cells after treatment with 24 inhibitors.

**
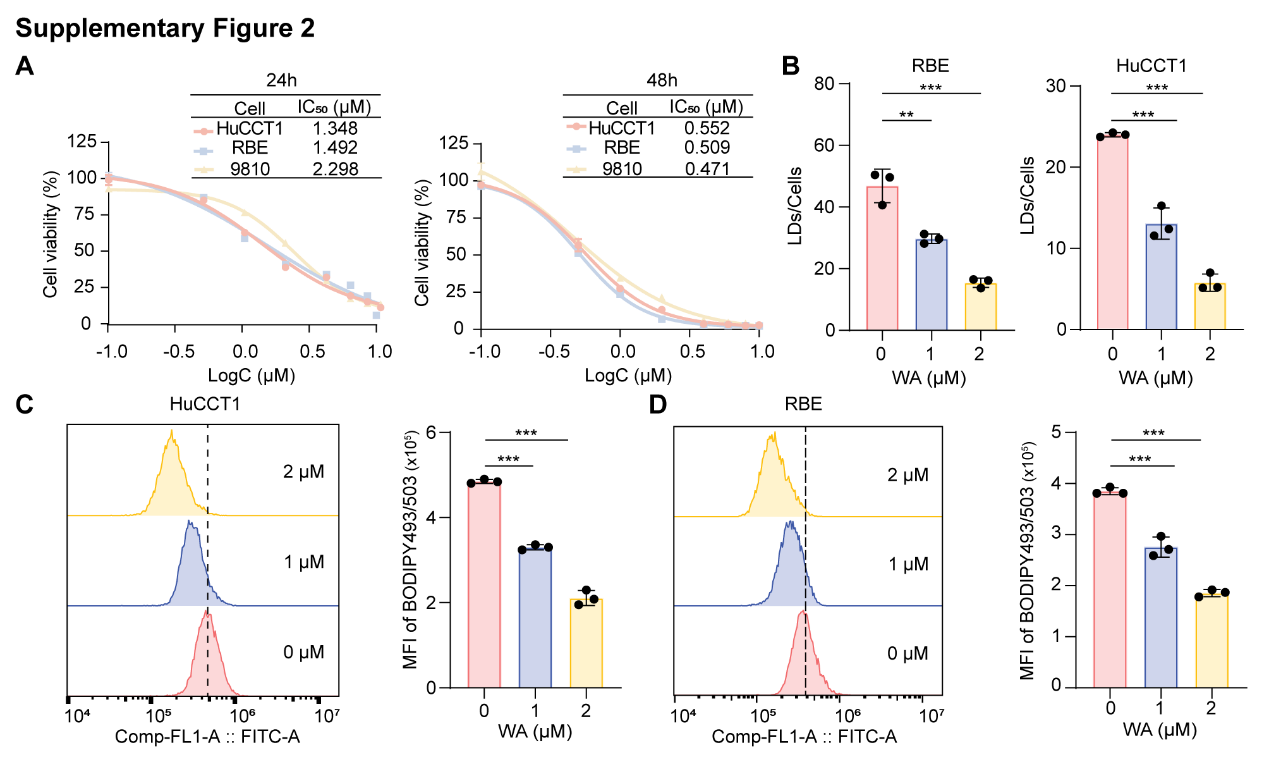
**

**Supplementary Figure 2.** (A) CCK-8 assay to determine the IC50 of three CCA cells, HuCCT1, RBE and HCCC-9810, treated with WA for 24 h and 48 h.(B) Quantitative analysis of lipid droplet content in BODIPY493/503 fluorescence staining and Oil red O staining. (C) BODIPY493/503 fluorescence staining and flow cytometry experiments were performed to quantify the lipid droplet content in WA-treated HuCCT1 cells. (D) BODIPY493/503 fluorescent staining and flow cytometry experiments were performed to quantify the lipid droplet content in WA-treated RBE cells. The data are represented as the mean ± SD of three independent experiments. * *P* < 0.05; ** *P* < 0.01; *** *P* < 0.001; ns, no significance.


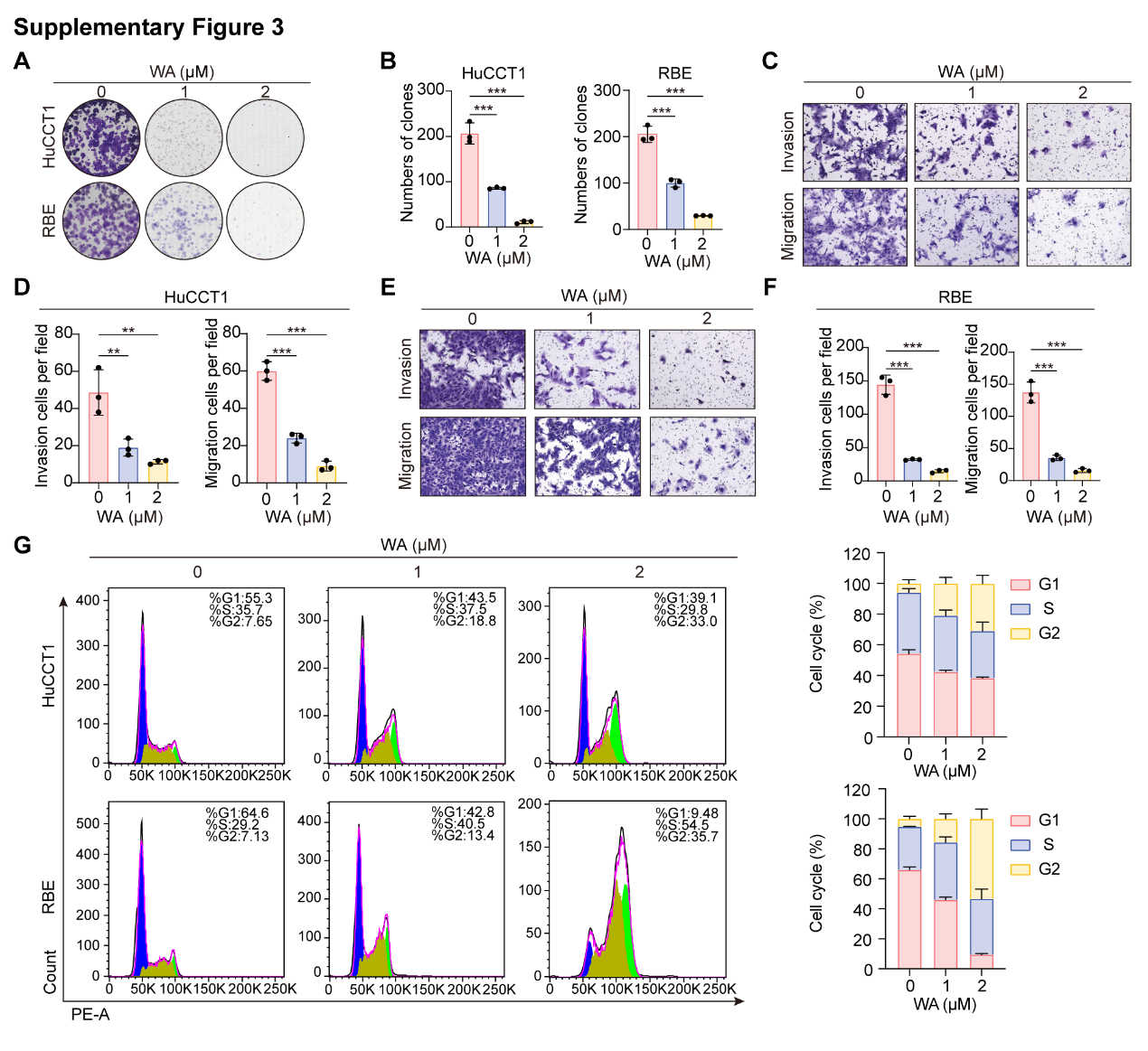


**Supplementary Figure 3.** (A) Clone formation assay to verify the effect of WA treatment on the proliferative capacity of HuCCT1 and RBE. (B) Quantitative analysis of clone formation assay. (C) Transwell assay to detect the effect of WA treatment on the migration and invasion ability of HuCCT1. (D) Quantitative analysis of HuCCT1 cell migration and invasion ability. (E) Transwell assay to detect the effect of WA treatment on the migration and invasion ability of RBE. (F) Quantitative analysis of RBE cell migration and invasion ability. (G) Flow cytometry assay to detect the cell cycle changes of HuCCT1 and RBE after WA treatment. The data are represented as the mean ± SD of three independent experiments. * *P* < 0.05; ** *P* < 0.01; *** *P* < 0.001; ns, no significance.

**
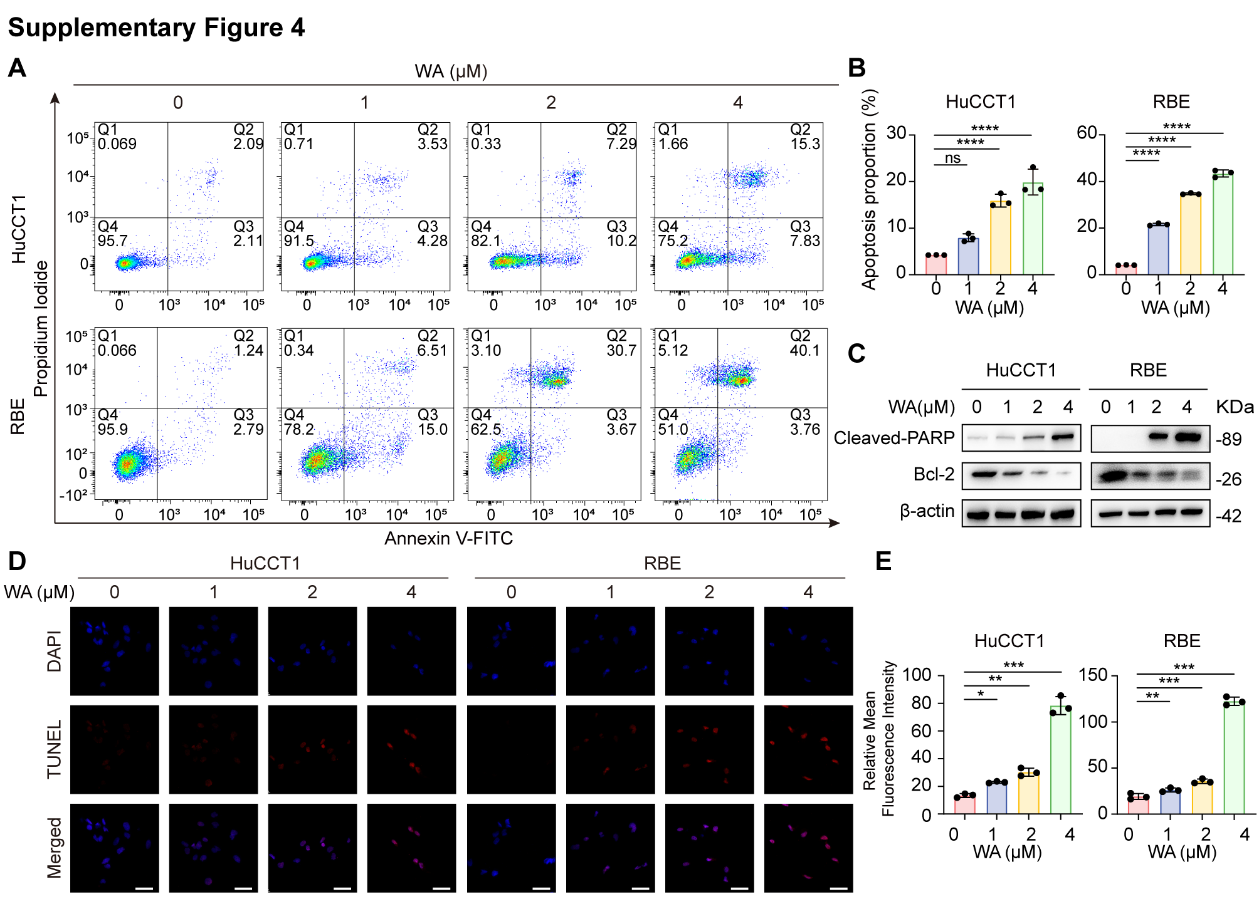
**

**Supplementary Figure 4.** (A) Flow cytometry assay to detect the apoptosis level of HuCCT1 and RBE after WA treatment. (B) Quantitative analysis of the proportion of apoptotic cells. (C) Western Blot assay to detect the expression levels of apoptosis-related proteins in HuCCT1 and RBE after WA treatment. (D) TUNEL assay to detect the apoptosis level of HuCCT1 and RBE after WA treatment, scale bar = 50 μm.(E) Quantitative analysis of TUNEL fluorescence intensity. The data are represented as the mean ± SD of three independent experiments. * *P* < 0.05; ** *P* < 0.01; *** *P* < 0.001; ns, no significance.


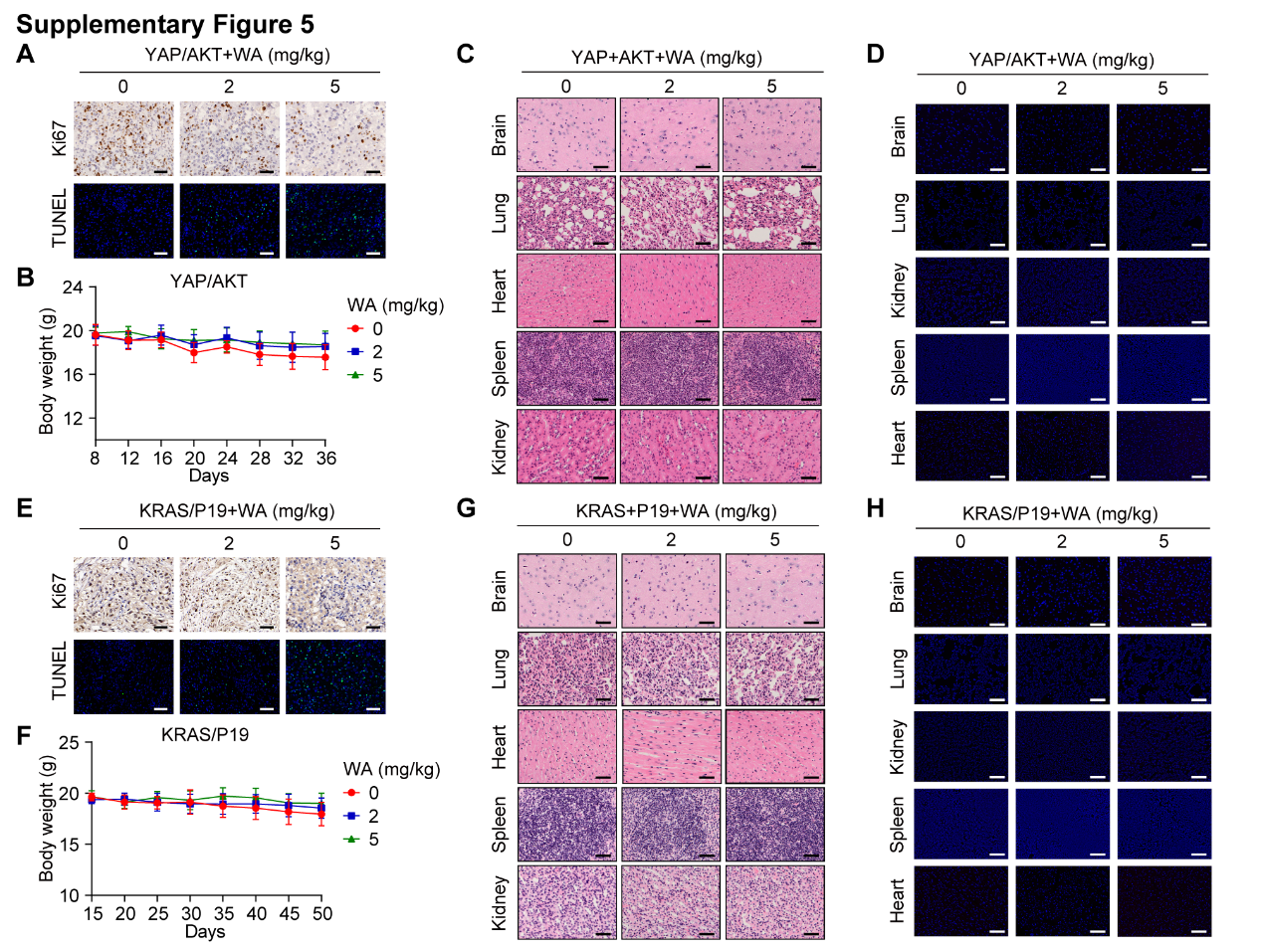


**Supplementary Figure 5**. (A) Ki67 and TUNEL staining to detect the effect of WA treatment in YAP/AKT model, scale bar = 50 μm. (B) Body weights of YAP/AKT model after treated with different dose of WA. (C) HE staining to detect the effect of WA treatment on the brain, heart, spleen, lung and kidney of mice in the YAP/AKT model, scale bar = 50 μm. (D) TUNEL staining to detect the effect of WA treatment on the brain, heart, spleen, lung and kidney of mice in the YAP/AKT model, scale bar = 20 μm. (E) Ki67 and TUNEL staining to detect the effect of WA treatment in KRAS/P19 model, scale bar = 50 μm. (F) Body weights of KRAS/P19 model after treated with different dose of WA. (G) HE staining to detect the effect of WA treatment on the brain heart, spleen, lung and kidney of mice in the KRAS/P19 model, scale bar = 50 μm. (H) TUNEL staining to detect the effect of WA treatment on the brain, heart, spleen, lung and kidney of mice in the KRAS/P19 model, scale bar = 20 μm.


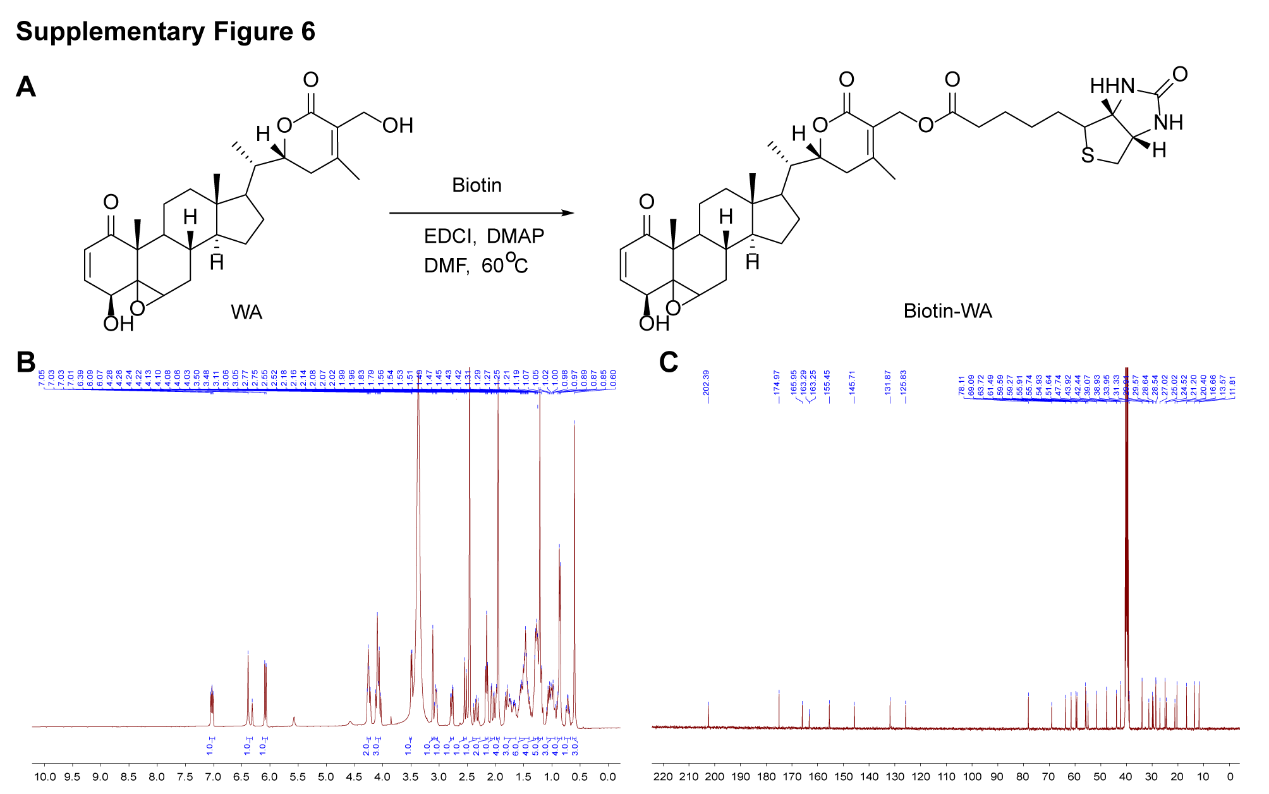


**Supplementary Figure 6**. (A) Synthesis of Biotin-WA probe. (B) ^1^H NMR spectrum of Biotin-WA. (C) ^13^C NMR spectrum of Biotin-WA.


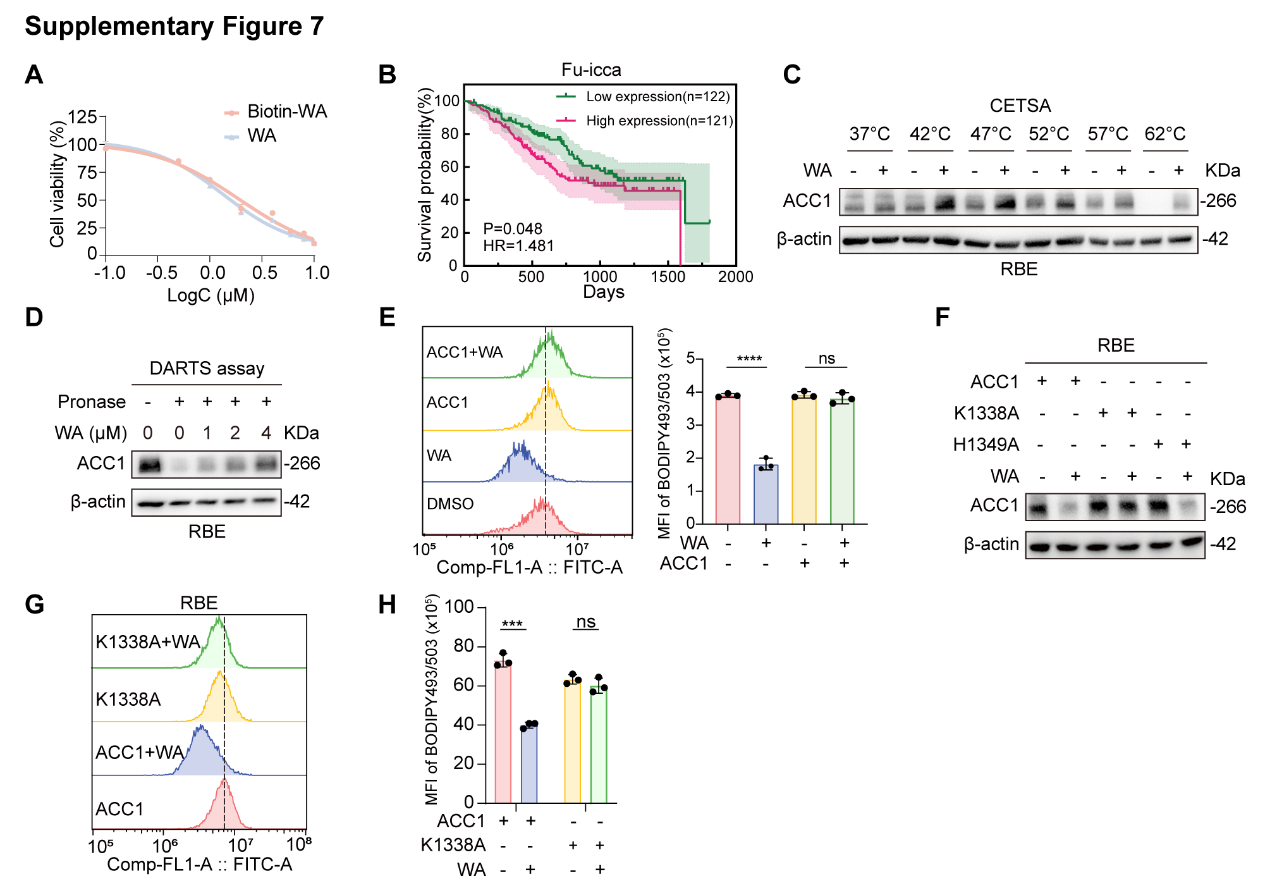


**Supplementary Figure 7.** (A) CCK-8 assay to detect the inhibitory effect of WA on CCA before and after biotin labeling. (B) Survival probability between ACC1^High^ and ACC1^Low^ groups in Fu-icca data sets. (C) CETSA assay to detect the interaction between WA and ACC1 in RBE. (D) DARTS assay confirmed that ACC1 is a direct target of WA in RBE. (E) Transfected RBE with ACC1 plasmids and treated with or without WA for 24 h. BODIPY493/503 fluorescence staining and flow cytometry experiments were performed to detect the content of lipid droplets in the cells. (F) Transfected RBE with K1338 or H1349 mutant plasmids and treated with or without WA for 24 h. Western Blot was performed to detect the expression level of ACC1. (G-H) RBE were treated with ACC1 WT or K1338 mutant plasmids and treated with or without WA for 24 h. BODIPY493/503 fluorescence staining and flow cytometry experiments were performed to detect the content of lipid droplets in the cells (G) and quantitatively analyzed (H). The data are represented as the mean ± SD of three independent experiments. * *P* < 0.05; ** *P* < 0.01; *** *P* < 0.001; ns, no significance.


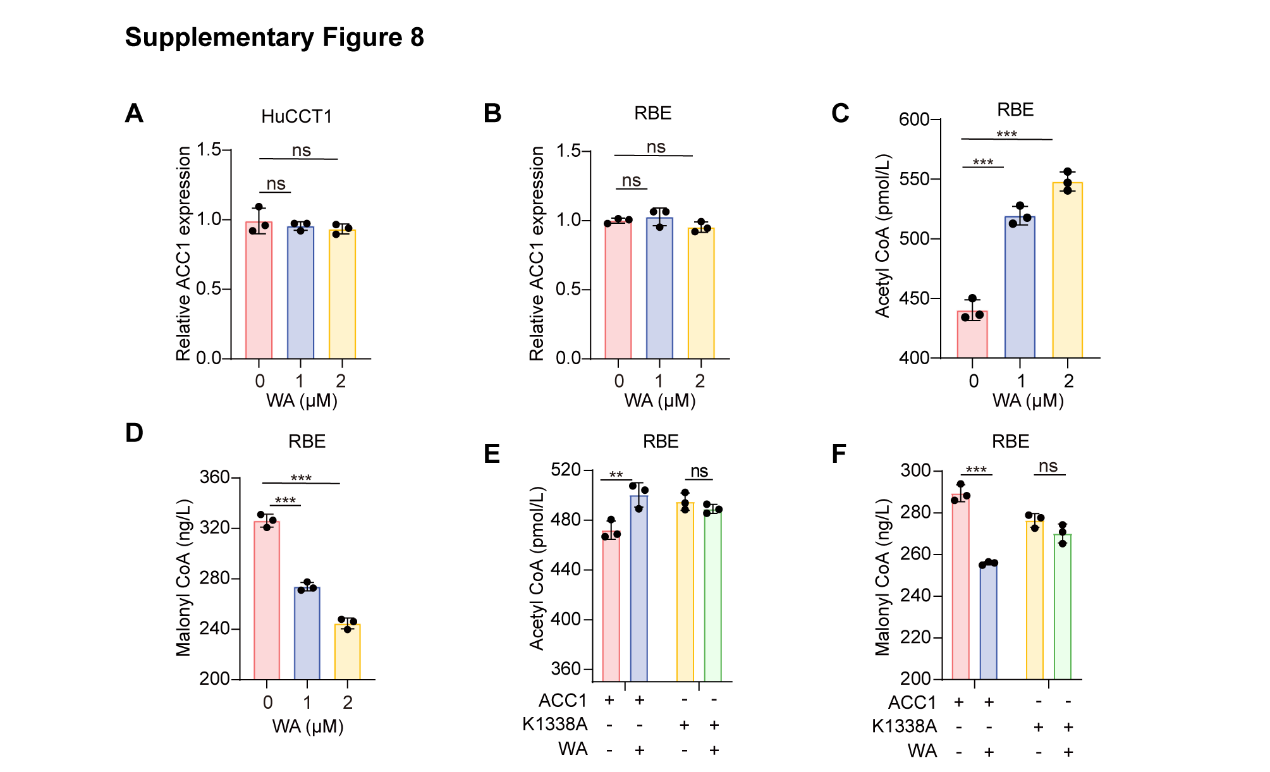


**Supplementary Figure 8.** (A) Detection of mRNA levels of ACC1 in HuCCT1 after WA treatment. (B) Detection of mRNA levels of ACC1 in RBE after WA treatment. (C-D) Detection of acetyl coenzyme A (C) and malonyl coenzyme A (D) levels in RBE after WA treatment using relevant kits. (E-F) RBE was transfected with wild-type ACC1 or K1338 mutant plasmid, the levels of acetyl coenzyme A (E) and malonyl coenzyme A (F) were detected after WA treatment. The data are represented as the mean ± SD of three independent experiments. * *P* < 0.05; ** *P* < 0.01; *** *P* < 0.001; ns, no significance.


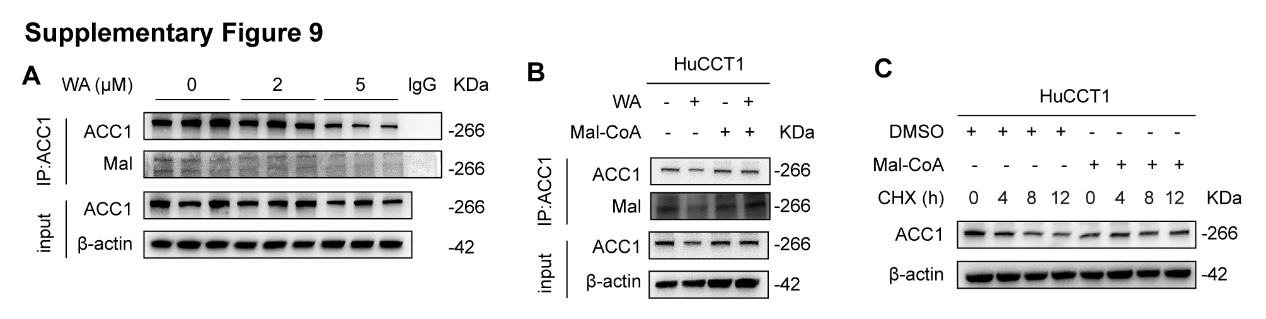


**Supplementary Figure 9.** (A) Detection of the ACC1 malonylation level of CCA mouse model after treated with different dose of WA by Co-IP assay. (B) Detection of the ACC1 malonylation level after Malonyl coenzyme A and WA treatment (2μM) by Co-IP assay. (C) Changes in the expression levels of ACC1 protein in HuCCT1 after CHX (100 μg/mL) treatment at different time points with or without supplementation with exogenous malonyl-CoA.


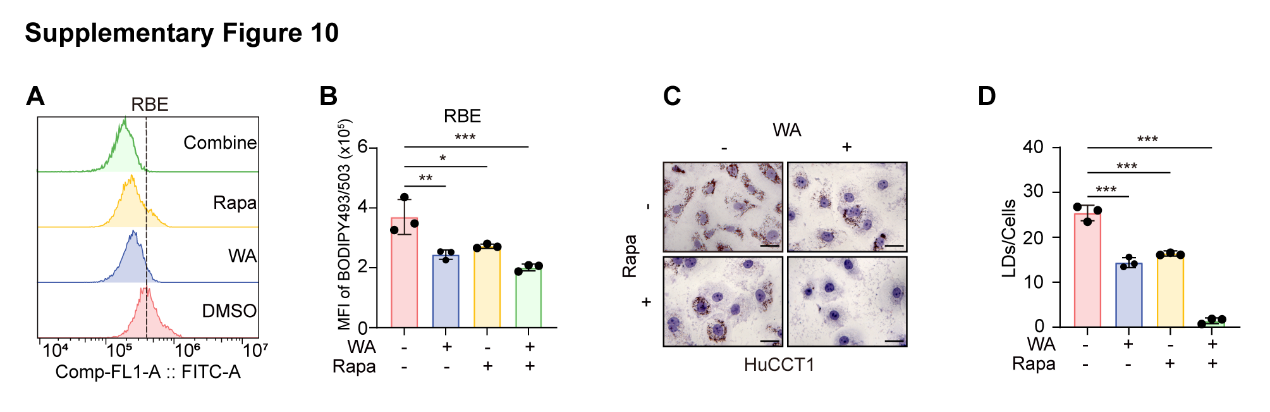


**Supplementary Figure 10.** (A-B) RBE were treated with WA or rapamycin, and lipid droplet content in the cells was detected and quantified by BODIPY493/503 fluorescent staining and flow cytometry experiments. (C-D) HuCCT1 were treated with WA or rapamycin, and lipid droplet content in the cells was detected and quantified by Oil red O staining assay, scale bar = 100 μm. The data are represented as the mean ± SD of three independent experiments. * *P* < 0.05; ** *P* < 0.01; *** *P* < 0.001; ns, no significance.
