# Supplementary material for: Pharmacological induction of acetyl-CoA carboxylase 1 autophagic degradation attenuates lipid accumulation and cholangiocarcinoma progression
Source: J Exp Clin Cancer Res. 2025 Nov 25;44:310. doi: 10.1186/s13046-025-03564-8 (PMC12645744; doi:10.1186/s13046-025-03564-8)
Supplement: Supplementary file 3 — Supplementary Material 3. [file 13046_2025_3564_MOESM3_ESM.pptx]

## Slide 1
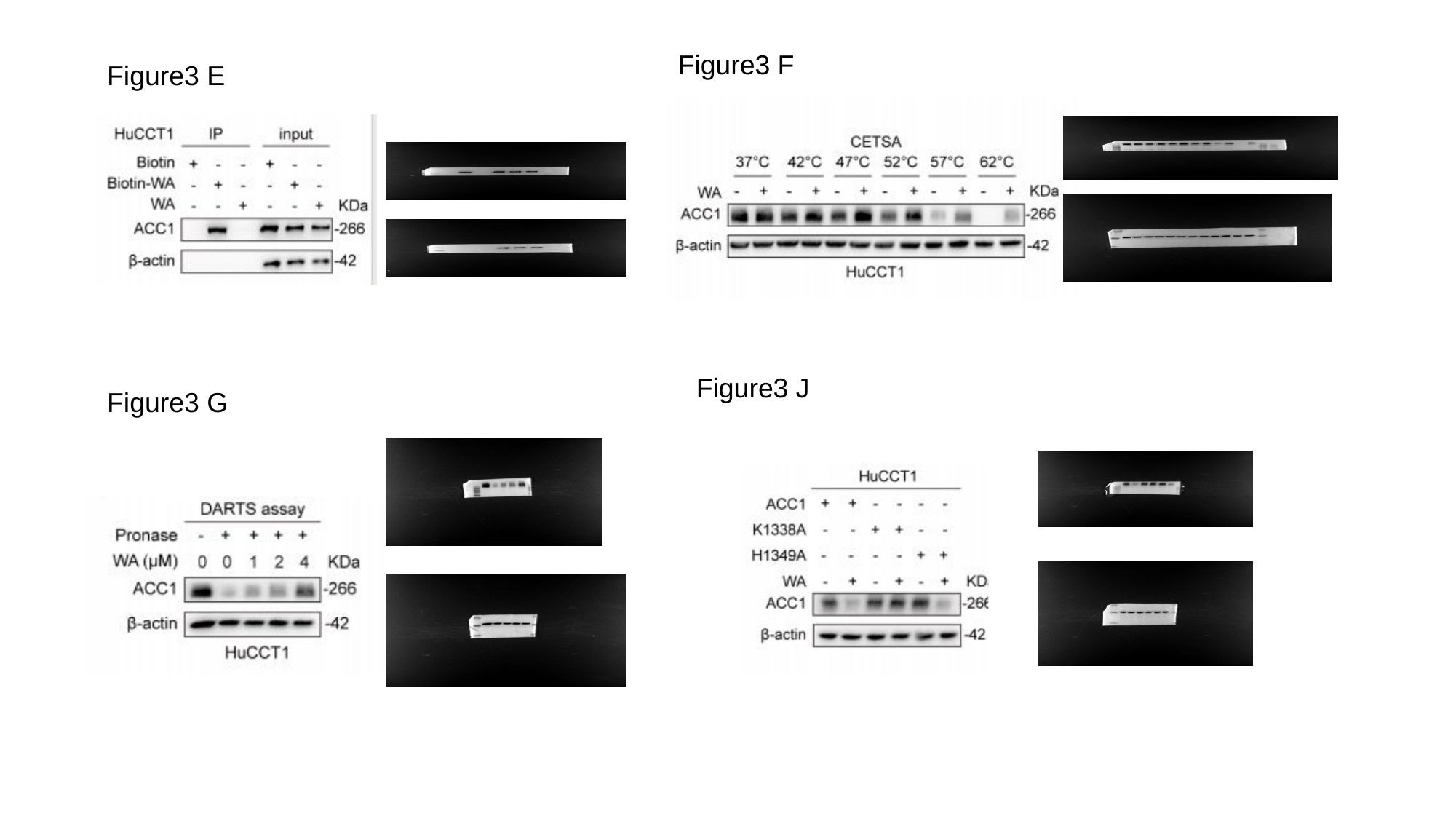

Figure3 F
Figure3 E
Figure3 J
Figure3 G

## Slide 2
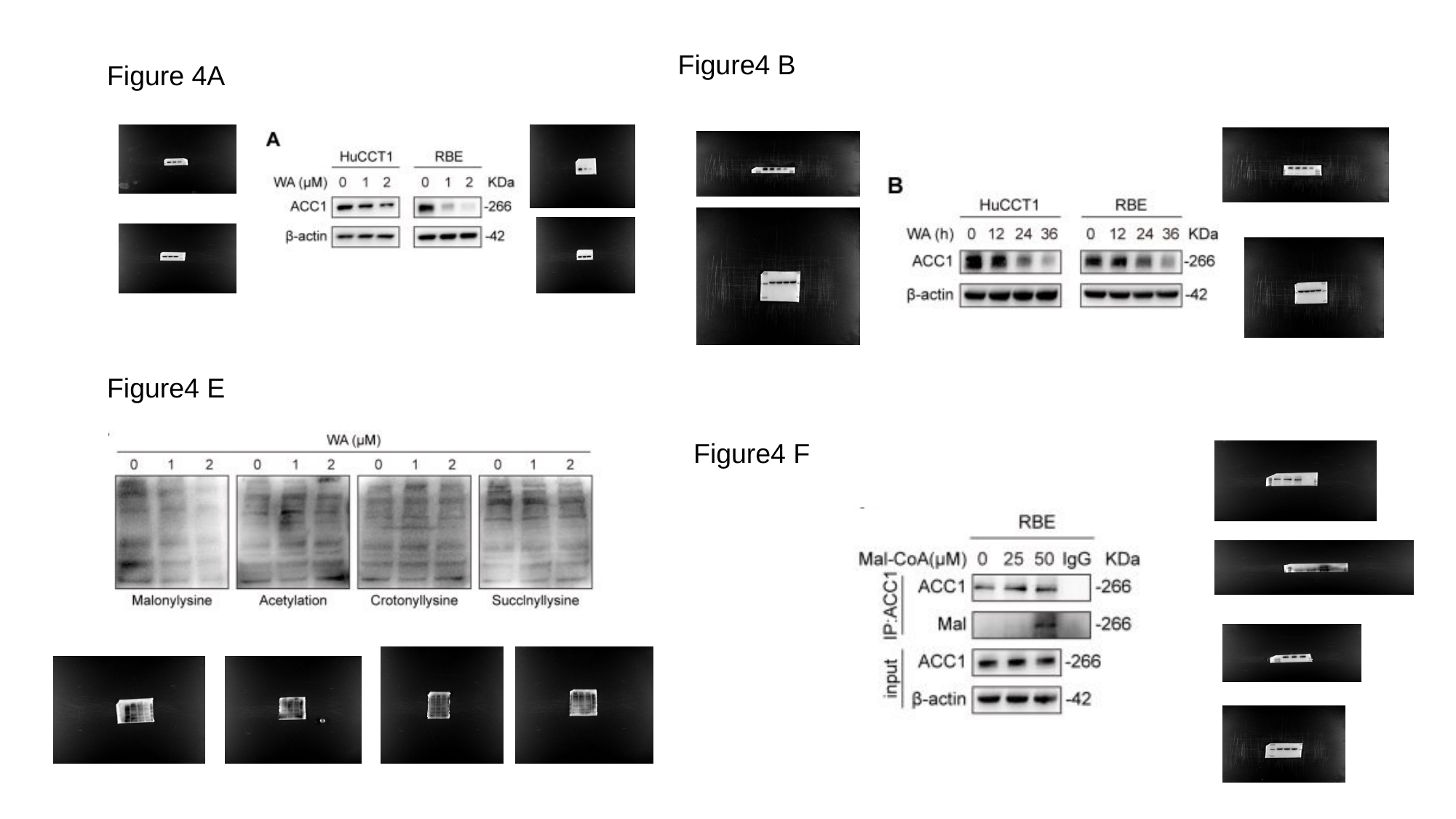

Figure4 B
Figure 4A
Figure4 E
Figure4 F

## Slide 3
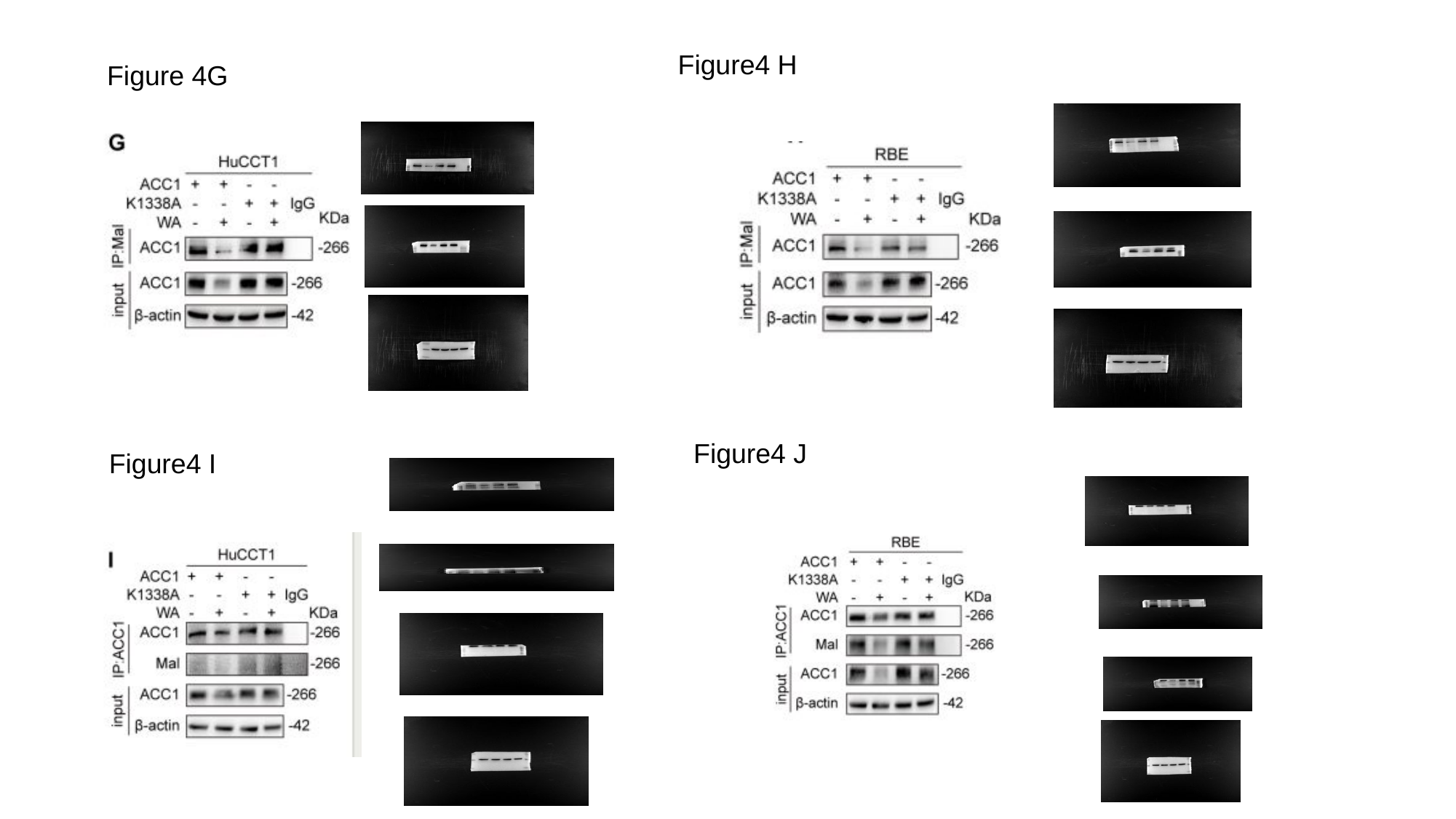

Figure4 H
Figure 4G
Figure4 J
Figure4 I

## Slide 4
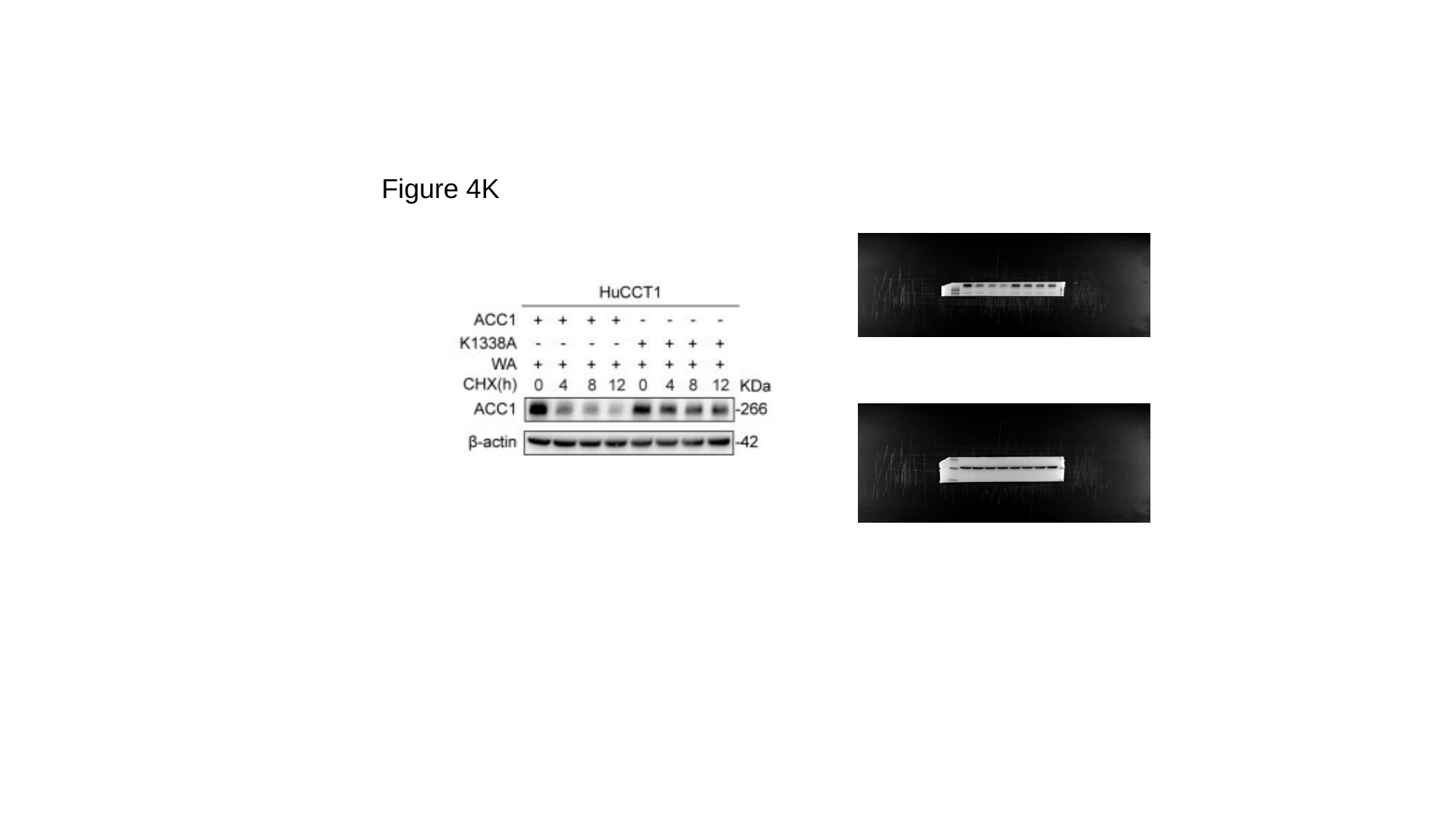

Figure 4K

## Slide 5
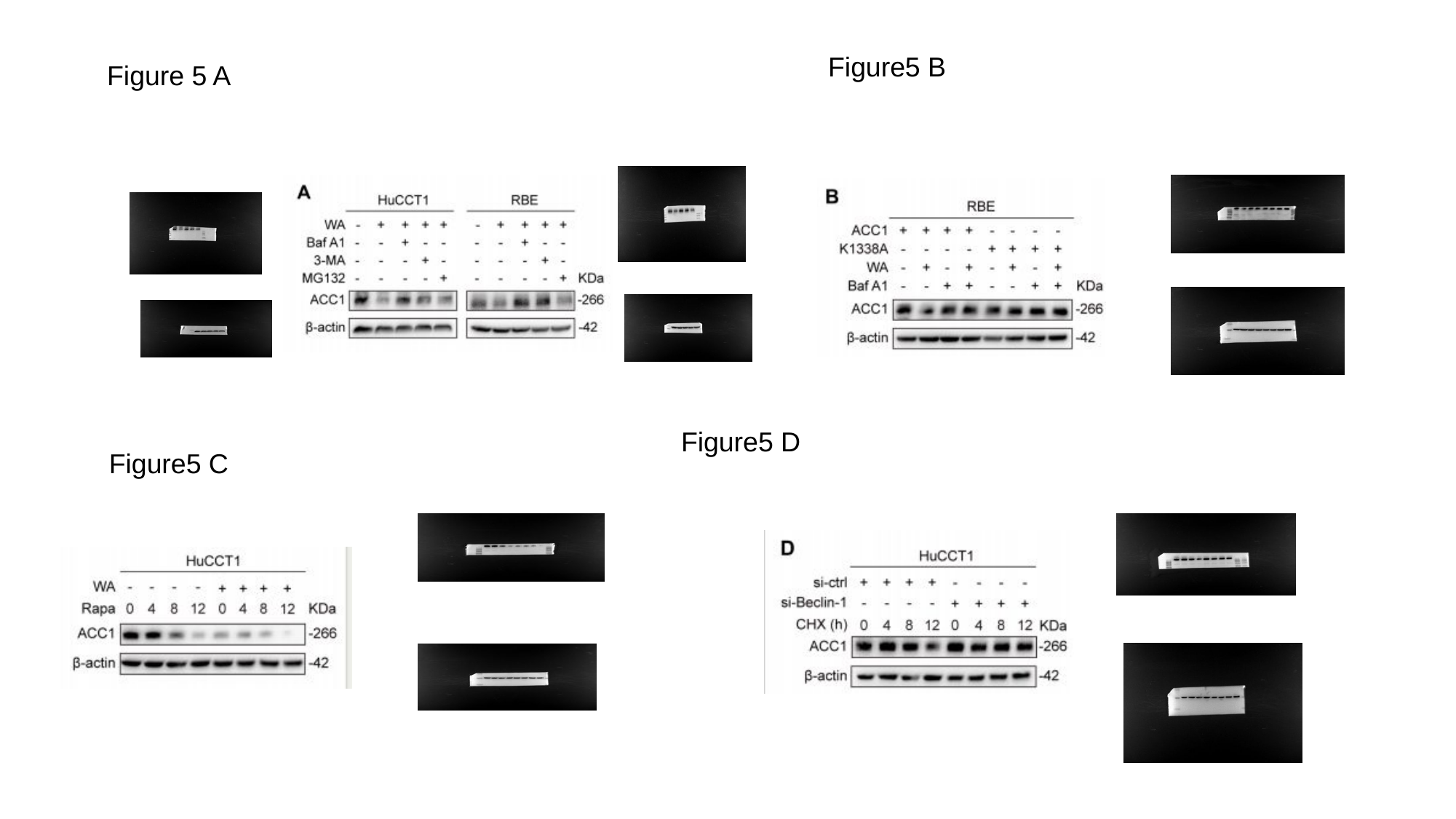

Figure5 B
Figure 5 A
Figure5 D
Figure5 C

## Slide 6
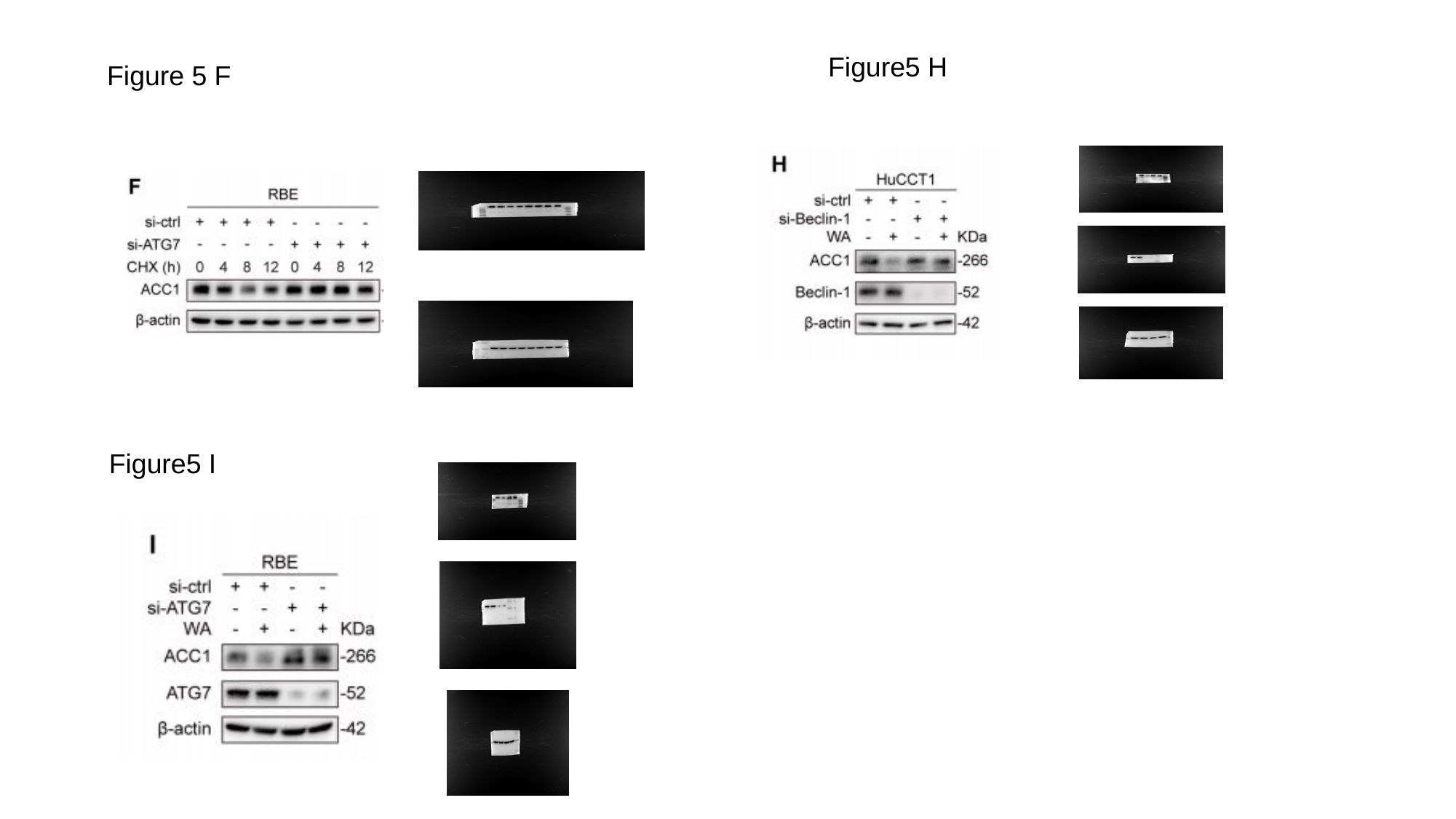

Figure5 H
Figure 5 F
Figure5 I

## Slide 7
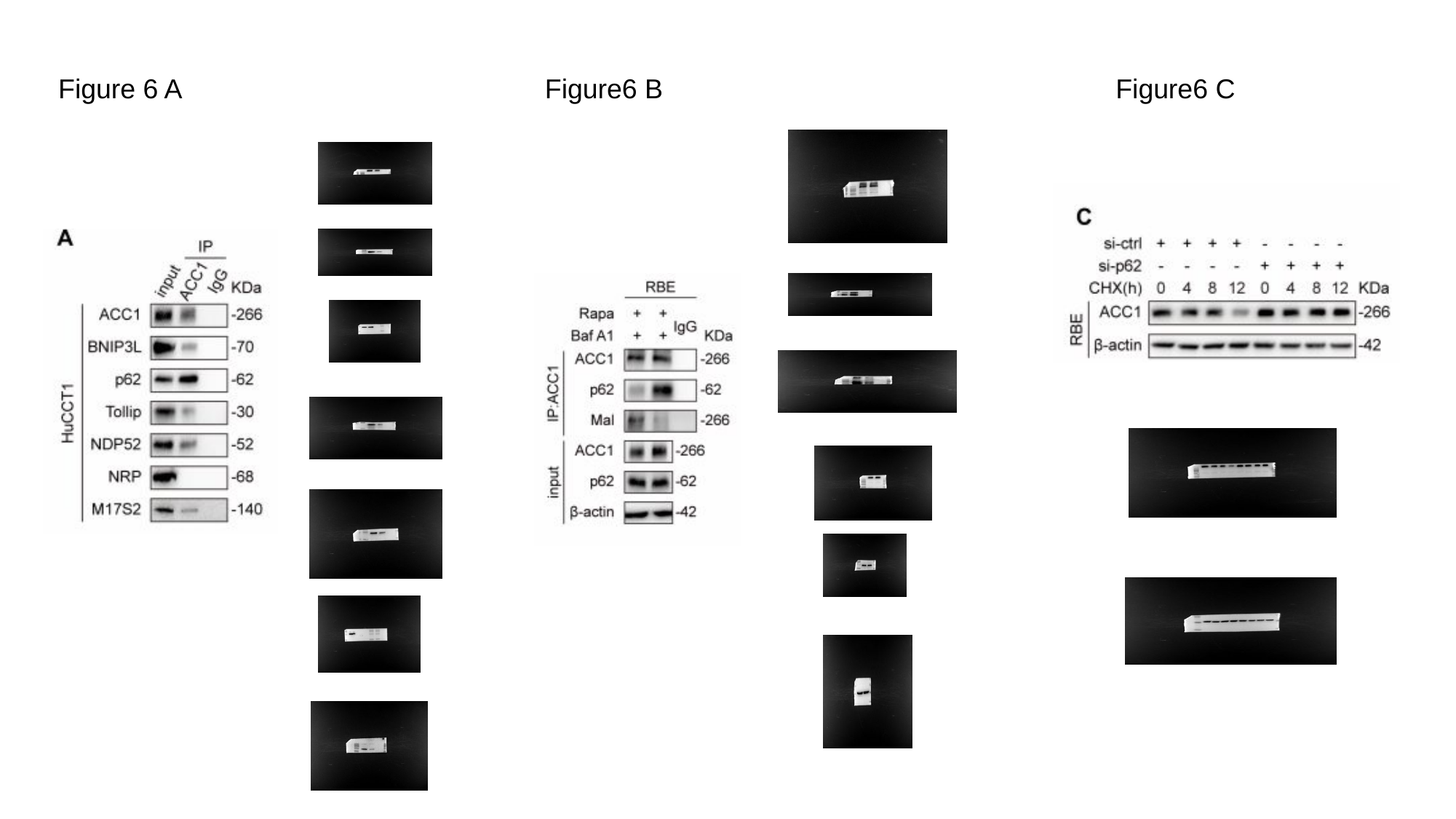

Figure 6 A
Figure6 B
Figure6 C

## Slide 8
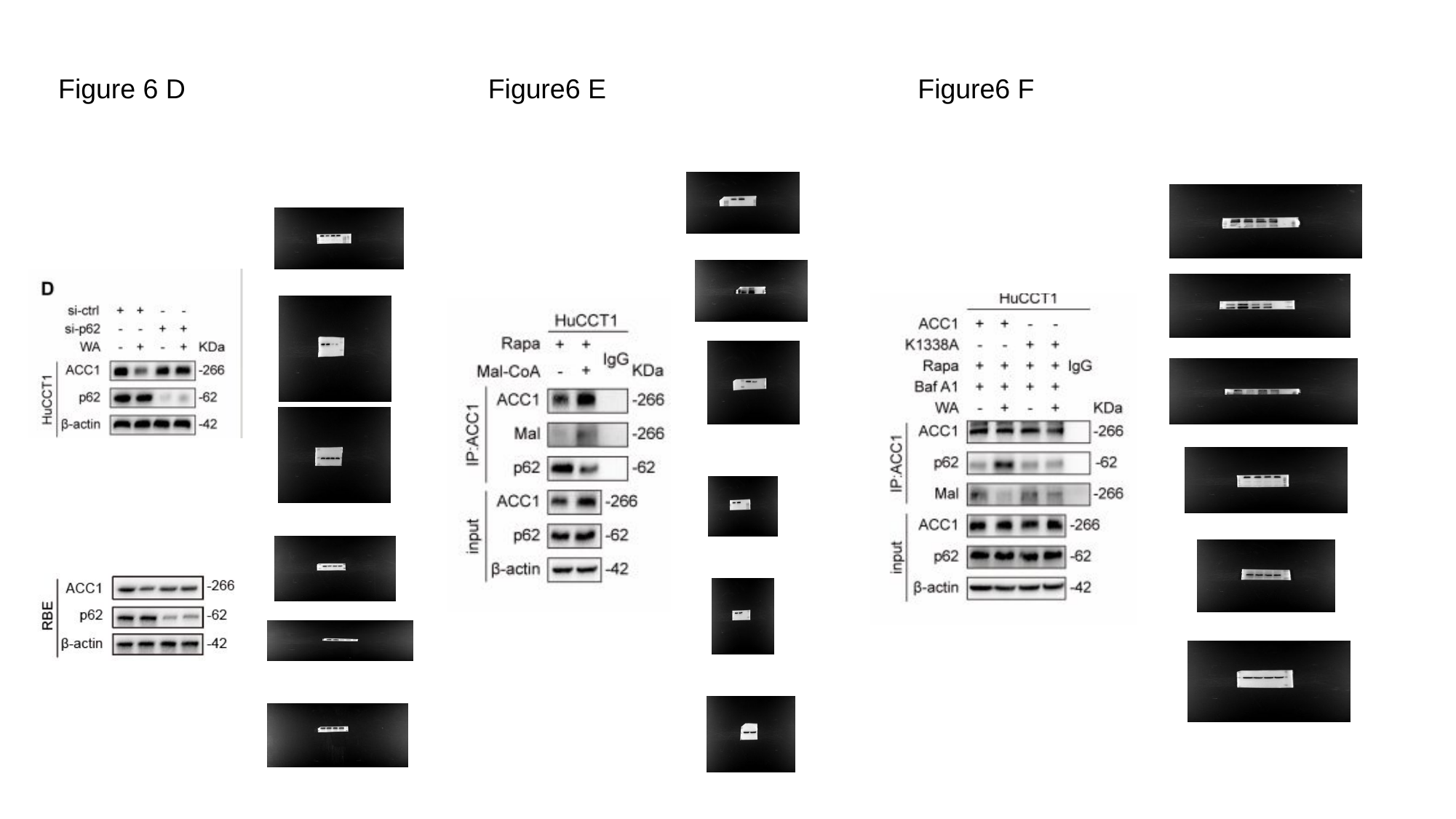

Figure 6 D
Figure6 E
Figure6 F

## Slide 9
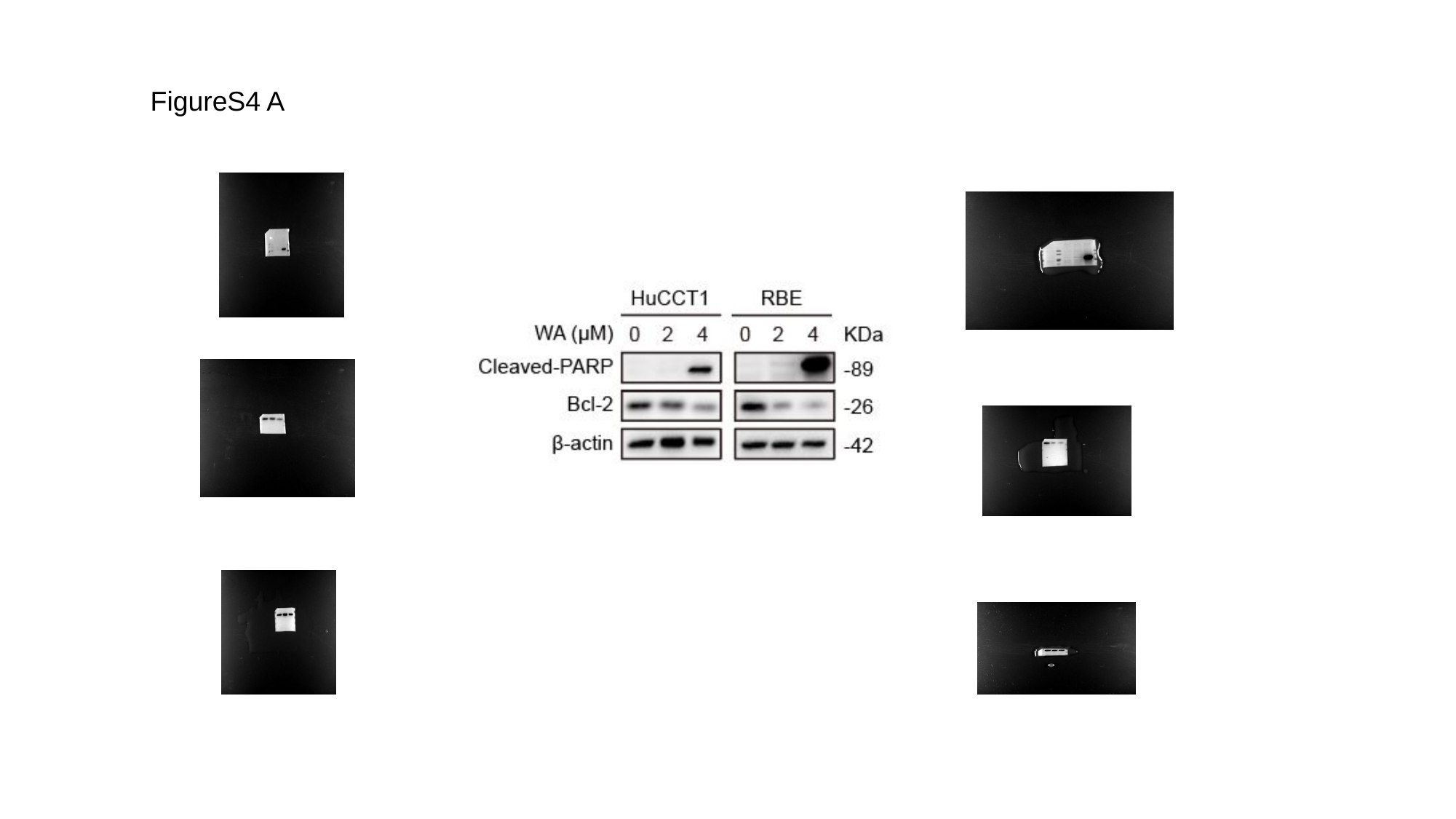

FigureS4 A

## Slide 10
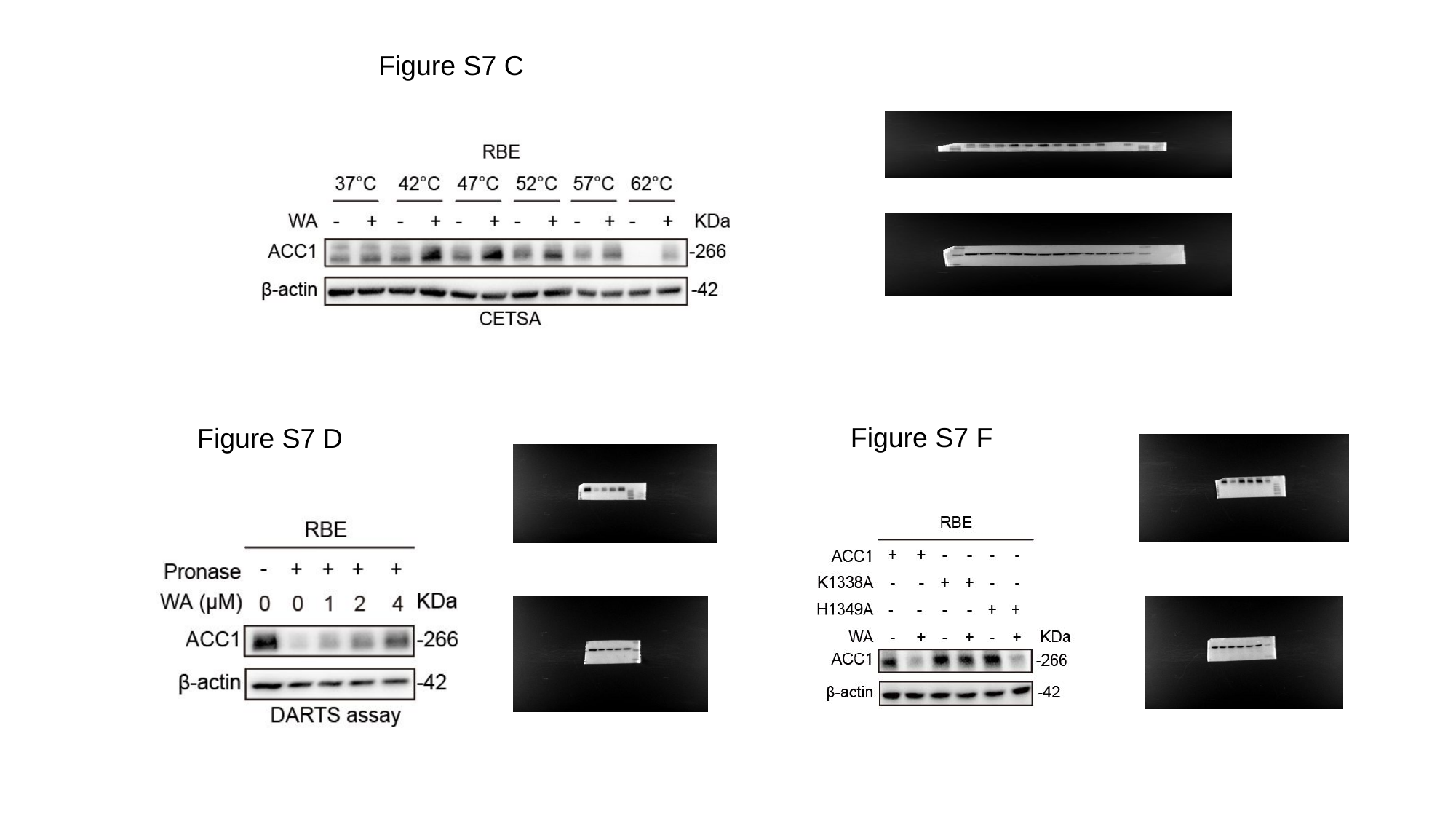

Figure S7 C
Figure S7 F
Figure S7 D

## Slide 11
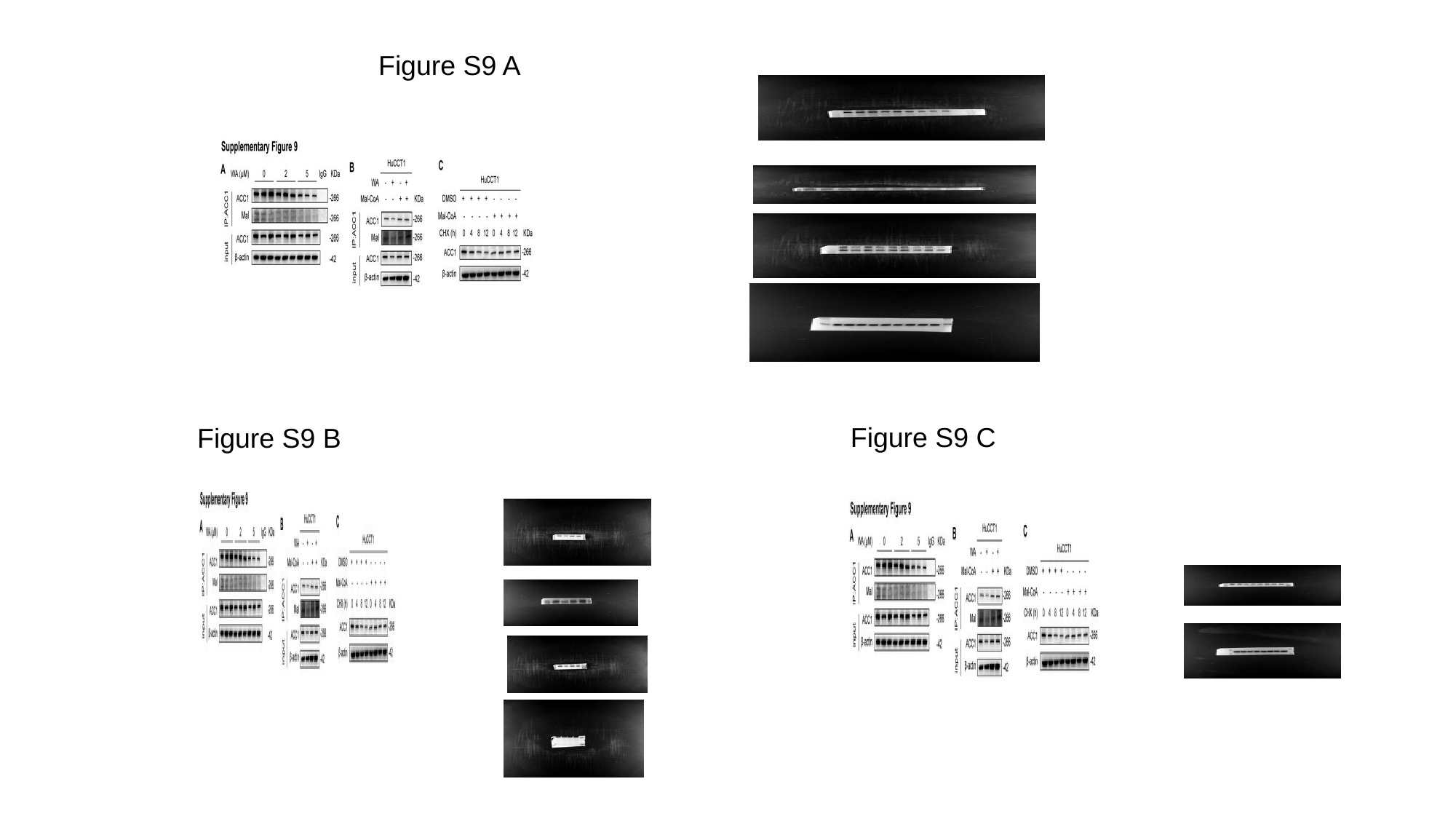

Figure S9 A
Figure S9 C
Figure S9 B
